# Hematological analysis of alpha-thalassemia: A single-center, retrospective clinical study

**DOI:** 10.1371/journal.pone.0329365

**Published:** 2025-08-04

**Authors:** Lin Zheng, Nuolan Yin, Meiying Wang, Hailong Huang, Na Lin, Shuyu Zhang, Linjuan Su, Liangpu Xu

**Affiliations:** 1 Department of Medical Genetic Diagnosis and Therapy Center, Fujian Maternity and Child Health Hospital, Fujian Key Laboratory for Prenatal Diagnosis and Birth Defect, Fuzhou, Fujian, China; 2 Department of School of Public Health, Fujian Medical University, Fuzhou, Fujian, China; Al Muthanna University, IRAQ

## Abstract

**Objectives:**

To determine the optimal cutoffs of the three indicators (MCV, MCH and HbA_2_) for alpha-thalassemia screening and to evaluate the validity of these indicators in Fujian Province, China.

**Methods:**

We conducted a retrospective analysis on the results of specimens received from May 2016 to April 2023. Receiver operating characteristic (ROC) curves were used to confirm the optimal cutoffs of the screening indicators. And the effectiveness of different combined screening methods was evaluated in patients with and without alpha-thalassemia.

**Results:**

The optimal cutoffs of MCV, MCH, and HbA_2_ were 77.85, 27.05 and 2.55, respectively. Among them, the area under the ROC curve of MCH was 0.912, and it was the best of the three parameters used for alpha-thalassemia screening.

**Conclusions:**

The results can help clinicians and laboratory technicians perform genetic counseling and prenatal diagnosis for patients. It also provide a reference for alpha-thalassemia genotype distributions in our region and the optimal cutoff values of MCV, MCH and HbA_2_.

## Introduction

Thalassemia is a prevalent hereditary hematologic disease all over the world [1], particularly in Southeast Asian countries [2–4]. The carrying rate of alpha-thalassemia is higher than that of beta-thalassemia [5–7] and is 3.17% in Fujian Province, China [8]. Alpha-thalassemia can lead to different degrees of anemia, and even hydrops fetalis syndrome [9], which has brought a heavy economic and spiritual burden to family and society. However, aside from bone marrow transplantation [10] and gene therapy [11–14], no effective treatment exists. Therefore, the detection of carriers and prevention of the birth of newborns with severe alpha-thalassemia through prenatal diagnosis were considered the most effective methods [15].

The confirmatory PCR test is relatively expensive and not available in many laboratories, especially in areas with limited resources. As a result, screening tests are important and used to decide who needed the PCR test. In practice, mean corpuscular volume (MCV), mean corpuscular hemoglobin (MCH) and hemoglobin A_2_ (HbA_2_) were the most commonly used to screen for alpha-thalassemia [15]. Herein, we conducted a large-scale survey to determine their optimal cutoffs for alpha-thalassemia screening in the city of southern China and evaluate the application value of combined screening based on these indicators.

## Methods

### Subjects and erythrocyte parameter detection

The present study followed the ethical principle and was approved by the Medical Ethics Committee of Fujian Maternity and Child Health Hospital, Affiliated Hospital of Fujian Medical University (No. 2021KLRD09016). All participants were informed and signed a written informed consent. All experiments were performed in accordance with the Declaration of Helsinki and National Regulations for Ethics of Biological Medical Sciences on Human Studies released by Ministry of Health, China. A total of 24647 participants who were admitted to our hospital from May 2016 to April 2023 were included in our study. Exclusion criteria included subjects who had no genotype test performed, those with other hemoglobinopathies, those with iron-deficiency anemia and those who had blood transfusion within one year. MCV and MCH were detected on an automated analyzer (XN3000; Sysmex, Japan).

Data for this retrospective study were first accessed for research purposes between June and July 2023. All personally identifiable information was removed prior to analysis, and the research team had no access to any identifiable patient data during or after data collection.

### Hemoglobin analysis

Hemoglobin was analyzed by an automated capillary electrophoresis analyzer (Capillarys2^TM^; Sebia, France). In addition to measuring the percentages of HbA_2_ and HbA, it can detect hemoglobin variants, such as hemoglobin H (HbH), hemoglobin constant spring (HbCS), and hemoglobin Barts (Hb Barts).

### Common genotype testing

DNA was extracted using the DNA Extraction Kit (Yaneng Biosciences, Shenzhen, China). The deletions (--^SEA^/, -α^4.2^/, and -α^3.7^/) and the non-deletions (α^CS^α/, α^QS^α/, and α^WS^α/) of alpha-thalassemia, and seventeen mutations of beta-thalassemia were identified by PCR-reverse dot-blot assay using commercial kits (Yaneng Biosciences, Shenzhen, China) as described previously [16]. All tests were performed according to the commercial kit instructions.

### --THAI and HKαα genotype testing

Genotyping for --^THAI^ was tested using gap polymerase chain reaction (gap-PCR), and HKαα genotype testing was performed by single PCR and nested PCR, as described previously [17,18].

### Statistical analysis

Data were analyzed using the Statistical Package for the Social Sciences (SPSS) version 24 (IBM Inc., Chicago, USA). The normal distribution of the variables was verified by the Kolmogorov-Smirnov test. The distribution of non-normal data was described in term of medians and percentiles. The Mann-Whitney U test was used to compare the differences in quantitative variables between the two groups. Receiver operating characteristic (ROC) curves were plotted and used to determine the best cutoffs of all variables. The area under the curve (AUC) was used to evaluate the diagnostic performances of the indicators.

## Results

**Fig 1:**
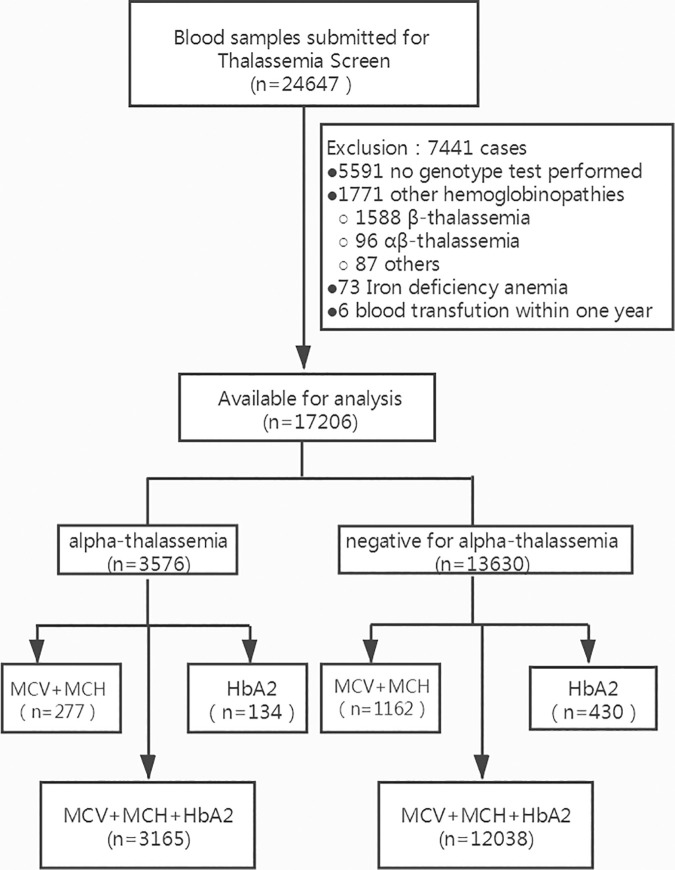
Distribution of samples according to current thalassemia screening pattern.

In all, 24647 individuals were recruited and tested, of whom 7441 were excluded from the study, including 5591 without thalassemia genotype result, 1771 with other hemoglobinopathies, 73 with iron deficiency anemia and 6 with blood transfusion within one year. 17206 were available for analysis. Among them, 13630 subjects with negative genetic results for alpha- and beta-thalassemia were designated as healthy group (4019 men and 9611 women; median age: 28.9 years). The other 3576 subjects with positive genetic results for alpha-thalassemia but negative for beta-thalassemia were categorized as alpha-thalassemia carriers (1371 men and 2205 women; median age: 29.4 years). In negative-for-alpha-thalassemia group, 12038 participants were tested for MCV, MCH and HbA_2_ simultaneously, 1162 participants were tested for MCVand MCH only, 430 participants were tested for HbA_2_ only. And they were 3165, 277 and 134 respectively in alpha-thalassemia group. These were shown in Fig 1. The genotypes were identified and are listed in Table 1.

**Table 1:** Genotypes identified among alpha-thalassemia carriers.

Genotype	Subjects(n)	Frequency(%)
**--** ^**SEA**^ **/αα**	2317	64.8
**-α** ^**3.7**^ **/αα**	754	21.1
**-α** ^**4.2**^ **/αα**	214	6.0
**α** ^**QS**^ **α/αα**	94	2.6
**α** ^**cs**^**α/αα**	71	2.0
**--** ^**THAI**^ **/αα**	34	1.0
**α** ^**ws**^**α/αα**	38	1.1
**--** ^**SEA**^ **/-α** ^**3.7**^	21	0.6
**--** ^**SEA**^ **/-α** ^**4.2**^	10	0.3
**-α** ^**3.7**^ **/-α** ^**3.7**^	5	0.1
**-α** ^**3.7**^ **/α** ^**QS**^ **α**	5	0.1
**--** ^**SEA**^ **/α** ^**ws**^ **α**	3	0.08
**-α** ^**3.7**^ **/-α** ^**4.2**^	2	0.06
**-α** ^**4.2**^ **/-α** ^**4.2**^	2	0.06
**-α**^**3.7**^**/α** ^**ws**^**α**	2	0.06
**--**^**SEA**^**/α** ^**cs**^**α**	1	0.03
**--** ^**SEA**^ **/ HKαα**	1	0.03
**-α**^**3.7**^**/α** ^**cs**^**α**	1	0.03
**α** ^**QS**^ **α/α** ^**QS**^ **α**	1	0.03
**Total**	3576	100

The results of the Kolmogorov-Smirnov test indicated that the distributions of the three indicators in the two groups were not normal. The data are described as the median (quartile) and are presented in Table 2. The three indicators were significantly lower in the alpha-thalassemia group than in the negative-for-alpha-thalassemia group based on the result of the Mann-Whitney U test (*P* < 0.05).

**Table 2:** Comparison of various parameters between two groups.

Indices/Groups	Alpha-thalassemia group	Negative-for-alpha-thalassemia group
**MCV (fl)**	70.9 (67.5, 78.8)	85.6 (81.2, 89.1)
**MCH (pg)**	22.1 (21.0, 25.9)	29.2(27.8, 30.4)
**HbA**_**2**_ **(%)**	2.4(2.2, 2.5)	2.6(2.5, 2.8)

The distribution of indices is described in the way of median (quartile).

**Fig 2:**
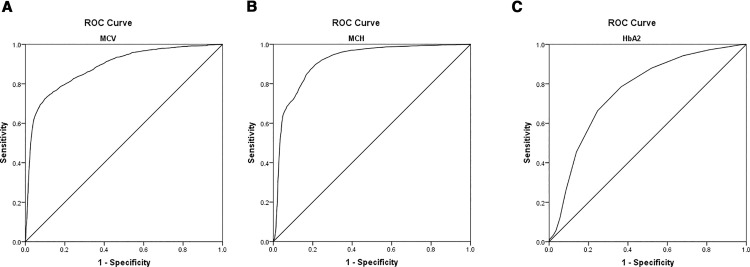
Receiver operating characteristic (ROC)curves for sensitivity and specificity of MCV(a), MCH(b) and HbA2(c) for alpha-thalassemia. MCV: Mean corpuscular volume; MCH: Mean corpuscular hemoglobin; HbA2: Hemoglobin A_2_.

The ROC curve analysis for alpha-thalassemia is shown in Fig 2 and Table 3. The AUC of MCV was 0.885 (*P* < 0.05, 95% CI was 0.878 ~ 0.891), and the best cutoff value to predict alpha-thalassemia was 77.85, giving a Youden index of 0.6249. The AUC of MCH was 0.912 (*P* < 0.05, 95% CI was 0.907 ~ 0. 918), and the best cutoff value to predict alpha-thalassemia was 27.05, giving a Youden index of 0.6841. The AUC of HbA_2_ was 0.761 (*P* < 0.05, 95% CI was 0.752 ~ 0.769), and the best cutoff value to predict alpha-thalassemia was 2.55, giving a Youden index of 0.4199.

**Table 3:** Analysis of ROC curves for three parameters for alpha-thalassemia.

Parameter	Cut-off value	Sensitivity	Specificity	FPR	FNR	+LR	-LR
**MCV**	77.85	0.7234	0.9015	0.0985	0.2766	7.3455	0.3068
**MCH**	27.05	0.8722	0.8120	0.1880	0.1278	4.6384	0.1574
**HbA**_**2**_	2.55	0.7851	0.6348	0.3652	0.2149	2.1499	0.3385

FPR-false positive rate, FNR-false negative rate, + LR-positive likelihood ratio, -LR-negative likelihood ratio.

Decision criteria of MCV/ MCH/HbA_2_ screening was positive for one item or more of MCV, MCH and HbA_2_, and MCV + MCH + HbA_2_ screening was positive for all of the three indices. Based on the cut-off value of these ROC curve analysis results, the comparison results of the schemes are shown in Table 4. The sensitivity of MCV/ MCH/HbA_2_ reached 95%. The genotypes of the patients who were missed by this scheme and the distribution of the three indicators in each group are shown in Table 5.

**Table 4:** Screening efﬁciencies of various parameters and their combinations.

Category	Se (%)	Sp (%)	PPV (%)	NPV (%)	Da (%)
**MCV/MCH/HbA**_**2**_	95.0	49.1	32.9	97.4	58.7
	(3007/3165)	(5914/12038)	(3007/9131)	(5914/6072)	(8921/15203)
**MCV + MCH + HbA**_**2**_	33.5	95.1	64.1	84.5	82.3
	(1061/3165)	(11445/12038)	(1061/1654)	(11445/13549)	(12506/15203)

Se, sensitivity, TP/(TP + FN) × 100%; Sp, specificity, TN/(FP + TN) × 100%; PPV, positive predictive value, TP/(TP + FP) × 100%; NPV, negative predictive value, TN/(FN + TN) × 100%, Da, diagnostic accuracy, (TP + TN)/(TP + FP + FN + TN) × 100%. The positive cases of alpha-thalassemia group were expressed with TP and the negative cases of alpha-thalassemia group were expressed with FN. The positive cases of negative-for-alpha-thalassemia group were expressed with FP and the negative cases of negative-for-alpha-thalassemia group were expressed with TN. MCV, mean corpuscular volume; MCH, mean corpuscular hemoglobin; HbA2: hemoglobin A_2_

**Table 5:** Various parameters among the cases missed by MCV + MCH + HbA_2_.

Genotype	Subjects(n)	MCV (fl)	MCH (pg)	HbA_2_ (%)
**-α** ^**3.7**^ **/αα**	113	83.7	28.2	2.70
**-α** ^**4.2**^ **/αα**	25	83.6	28.0	2.70
**α** ^**ws**^**α/αα**	16	82.9	28.6	2.81
**--** ^**SEA**^ **/αα**	3	82.6	27.4	2.73
**α** ^**cs**^**α/αα**	1	85.1	28.0	2.60

The distribution of indices is described in the way of median.

## Discussion

Thalassemia is a common monogenic diseases in the world [19–21]. Within our present clinical practice, individuals exhibiting reduced levels of MCV, MCH, and HbA2 underwent additional genetic assessments. It was necessary to confirm the optimal cutoffs of these indicators. In our ROC curve analysis, the optimal cutoffs of MCV, MCH and HbA_2_ were 77.85 fL, 27.05 pg and 2.55%, respectively. Among them, the cutoff of MCH and HbA_2_ were very close to clinical application, but that of MCV was different from the current commonly used clinical criteria (80 fL). Although screening test required relatively high sensitivity, the excessive genetic testing caused by false positives can impose certain financial burdens on patients and society. In this study, there were 1006 individuals (accounting for 7.6%) whose MCV values were between 77.85 and 80 fL in the negative-for-alpha-thalassemia group. The specificity of 82.53% using 80 fL as a cutoff value was lower than that of 90.15% using 77.85 fL as a cutoff value. This result indicated that unnecessary genetic testing would be avoided because of false-positive screening results if the optimal cutoff of 77.85 fL was adopted.

Of the three indicators, the AUC of HbA_2_ was 0.761, which showed moderate diagnostic value for alpha-thalassemia. According to the optimal cutoff, the specificity of HbA_2_ was 63.48%, which was lower than that of the other indicators. The sensitivity and false-positive rate were 78.51% and 36.52%, and the positive likelihood ratio was 2.1499, indicating that the positive screening results of HbA_2_ had a poor effect on the diagnosis of alpha-thalassemia. Additionally, some internal medical conditions had an impact on the accuracy of HbA_2_ [22]. In contrast, the AUC and Youden index of MCH were 0.912 and 0.6841, respectively, which were significantly higher than those of MCV and HbA_2_, indicating that this parameter had a higher diagnostic value for alpha-thalassemia. The sensitivity was 87.22%, and the false-negative rate was 12.78%, indicating a low rate of missed diagnoses of alpha-thalassemia. Moreover, this study found that those individuals with normal MCH in the alpha-thalassemia group mainly had mild alpha-thalassemia. Therefore, the indicator MCH should be given adequate attention in clinical practice, especially in alpha-thalassemia high-prevalence areas. Furthermore, MCH exhibits greater stability in stale blood specimens than MCV [23], and it is less affected by physiological factors and drugs [24]. Thus, from this, MCH is the best among the three parameters. At present, the incidence of alpha-thalassemia is consistently high because of the lack of standardized prevention system in China. Although regular blood transfusion and iron removal [25–28] can control the progression of alpha-thalassemia in the short term, the most effective preventive measure is still to prevent the birth of fetuses with alpha-thalassemia major [1,29] by carrier screening and prenatal diagnosis. The cutoffs we have studied was based on a large amount of sample data. According to the optimal cutoff value obtained by ROC curve analysis, the sensitivity of the combined screening of MCV/MCH/HbA_2_ reaches 95%, while the specificity of the screening strategy of MCV + MCH + HbA_2_ can reach 95.1%. In the 3165 alpha-thalassemia carriers, 158 cases (accounting for 5%) had negative results for all three indicators simultaneously. Among them, 113 cases had -α^3.7^/αα, 25 cases had -α^4.2^/αα, 16 cases had α^ws^α/αα, 3 cases had --^SEA^/αα, and 1 case had α^cs^α/αα which was identified as thalassemia due to the appearance of the cs band during the detection of HbA_2_ by capillary electrophoresis. Moreover, all these cases were silent and mild alpha-thalassemia. After followed-up, none of them showed any symptoms of anemia and had no history of blood transfusion. However, if both parents were carriers of mild alpha-thalassemia, it was possible to give birth to a fetus with severe alpha-thalassemia. Therefore, for prenatal screening, stricter control of the screening evaluation criteria was necessary. In a word, it was recommended to use different evaluation criteria according to different clinical needs.

Some genotypes may show specific electrophoretic bands (such as HbH, HbBarts and CS band) which also suggested alpha-thalassemia. Therefore, the actual detection rate of the combined screening method will be higher than the statistical data in this study. On the other side capillary electrophoresis is helpful for the detection of some abnormal hemoglobins, such as Hb New York, Hb Q-Thailand, Hb J-Bangkok. In this sense, although the diagnostic value of HbA_2_ is lower than that of MCV and MCH, hemoglobin analysis is still necessary. In addition, common genetic tests may miss rare and novel thalassemia genotypes. In this study, through the above screening indicators, we identified a special type of alpha-thalassemia that was negative for conventional genetic testing. Its genotype was a mutation at codons 90−93 (−8 bp) (-AGCTTCGG), which was the first case discovered in the Fujian region [15]. In summary, the simultaneous screening of alpha-thalassemia using hematologic parameters and hemoglobin analysis in the prevalent region is recommended.

Although this study was a single-center retrospective investigation, as the prenatal diagnosis center of our province, we have enough cases due to referrals from the sub-centers in various cities. Therefore, the results of this study can largely represent the situation in Fujian region. The main limitation of this study was the incompleteness of the data because of the retrospective analysis. Of 24647 participants, 5591 subjects with negative initial screening or other reasons did not undergo further genetic testing, resulting in a loss of data. This may cause a deviation in the prevalence rate of the disease. Furthermore, due to the small number of individuals with double heterozygous alpha-thalassemia in this study, it is difficult to analyze whether there were significant differences in these parameters between alpha-thalassemia carriers and individuals with double heterozygous alpha-thalassemia mutations. The latter could potentially aid in estimating the likelihood of heterozygous versus homozygous alpha thalassemia in parents, particularly in cases where genetic mutations cannot be definitively identified. This distinction may provide a basis for clinical decision-making regarding whether chorionic villus sampling (CVS) testing should be conducted during prenatal diagnosis. This has been one of the most challenging scenarios in the prenatal diagnosis of alpha thalassemia. Further research will be necessary in the future. In short, the results of this study provide a reference for local clinicians for more useful clinical consultation. At the same time, the results will also be helpful for laboratory professionals as a basis for more accurate reference ranges in the region.

## Supporting information

S1 Fig Raw images of alpha-thalassemia gene detection by PCR-reverse dot-blot assay. (ZIP)

S2 Fig Representative chromatograms from hemoglobin electrophoresis. (ZIP)

S1 Table Hematological parameters of carriers with alpha-thalassemia. (XLSX)

S2 Table Hematological parameters of Negative-for-alpha-thalassemia group. (XLSX)
