# Supplementary figures and images for: Hematological analysis of alpha-thalassemia: A single-center, retrospective clinical study
Source: PLoS One. 2025 Aug 4;20(8):e0329365. doi: 10.1371/journal.pone.0329365 (PMC12321134; doi:10.1371/journal.pone.0329365)

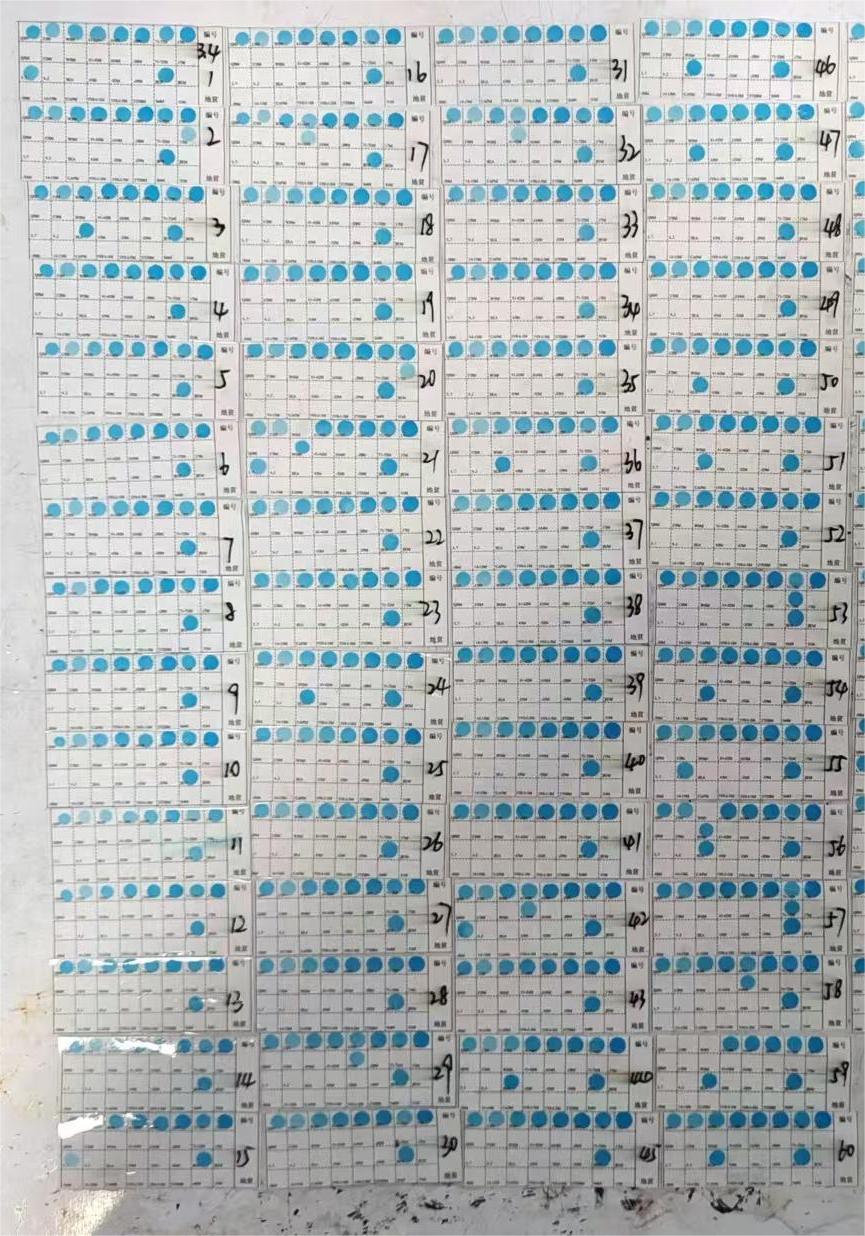

Supplement: S1 Fig — (ZIP) [file pone.0329365.s001.zip › S1_Fig1.jpg]

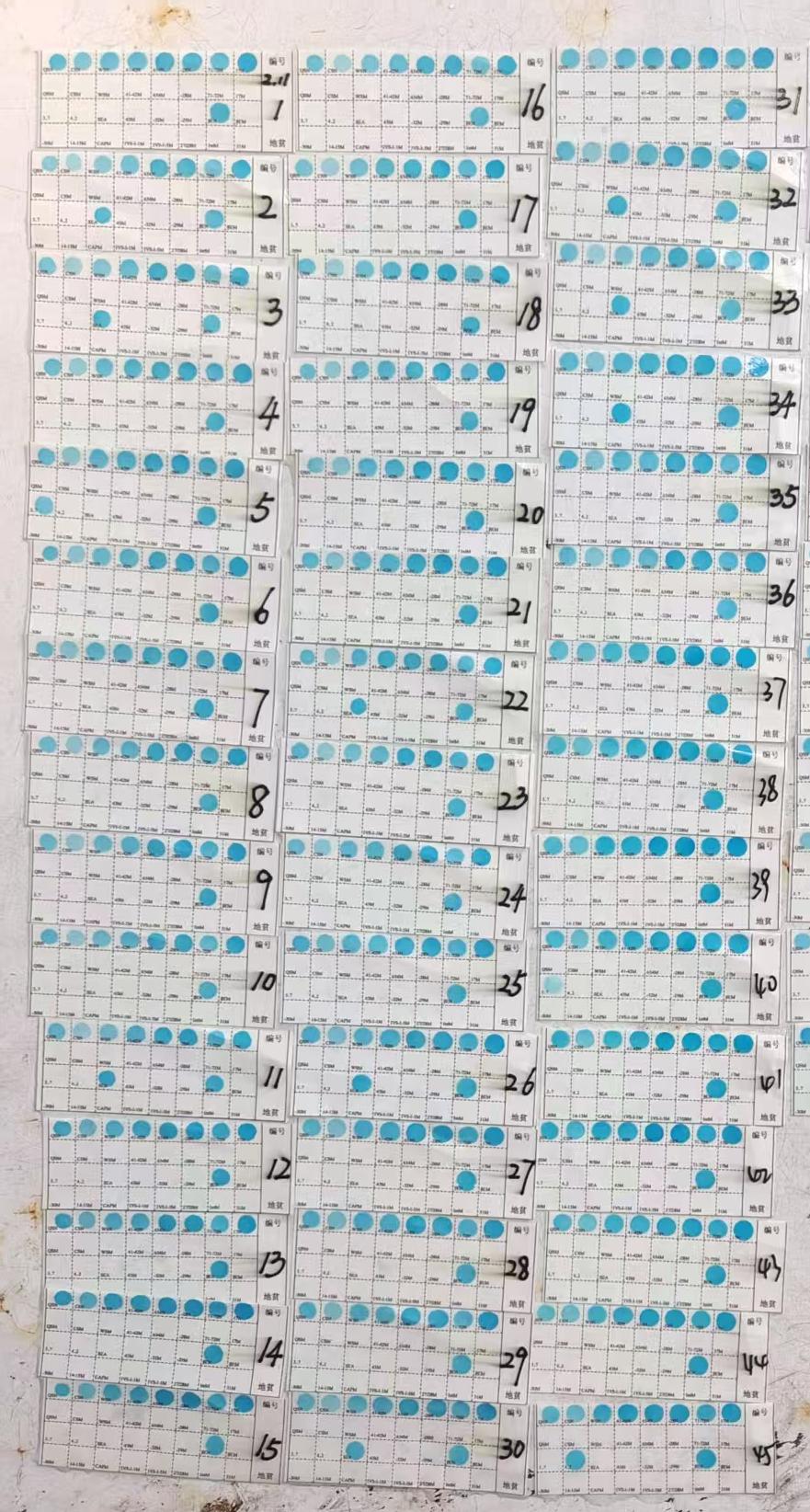

Supplement: S1 Fig — (ZIP) [file pone.0329365.s001.zip › S1_Fig10.jpg]

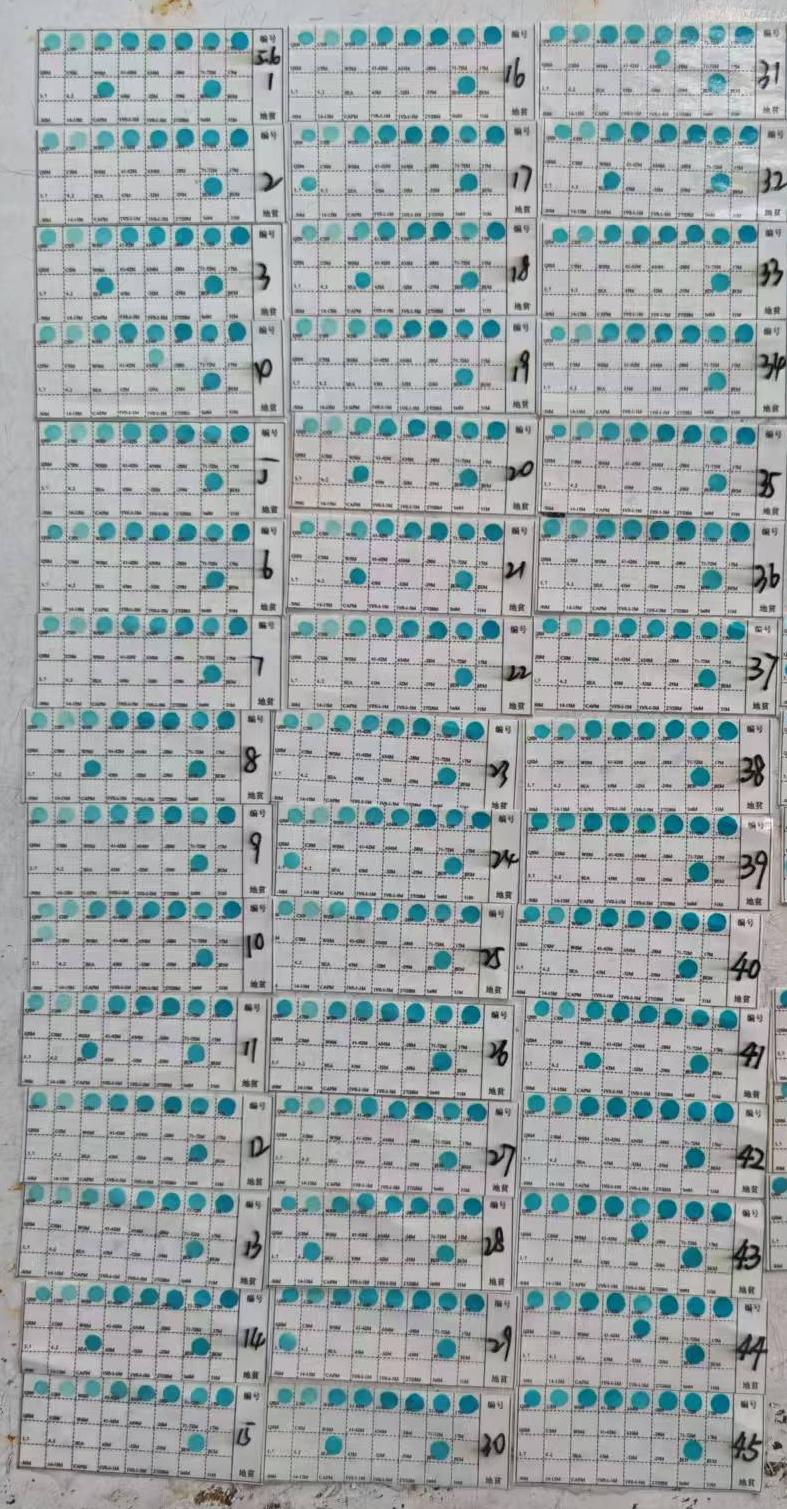

Supplement: S1 Fig — (ZIP) [file pone.0329365.s001.zip › S1_Fig11.jpg]

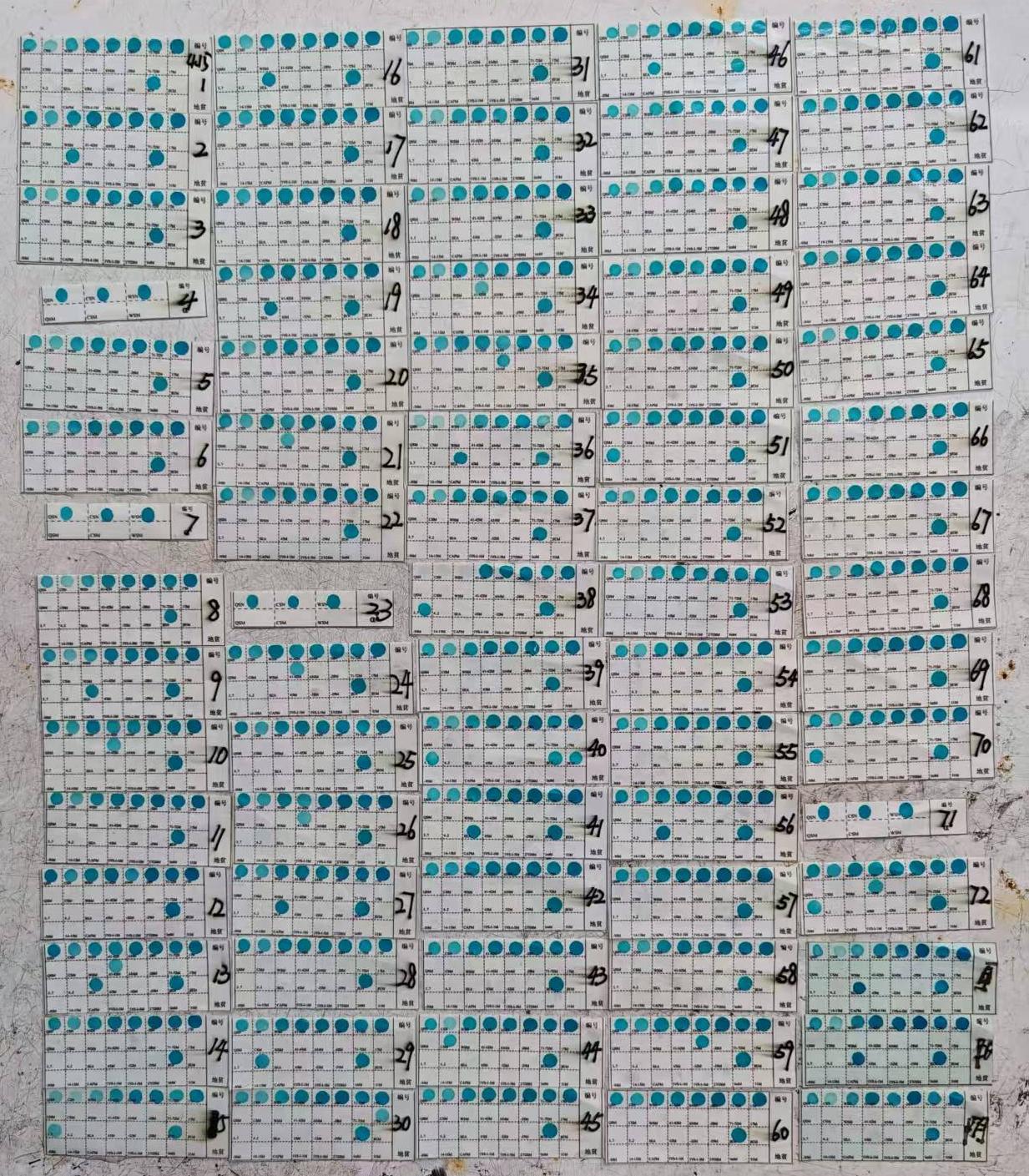

Supplement: S1 Fig — (ZIP) [file pone.0329365.s001.zip › S1_Fig12.jpg]

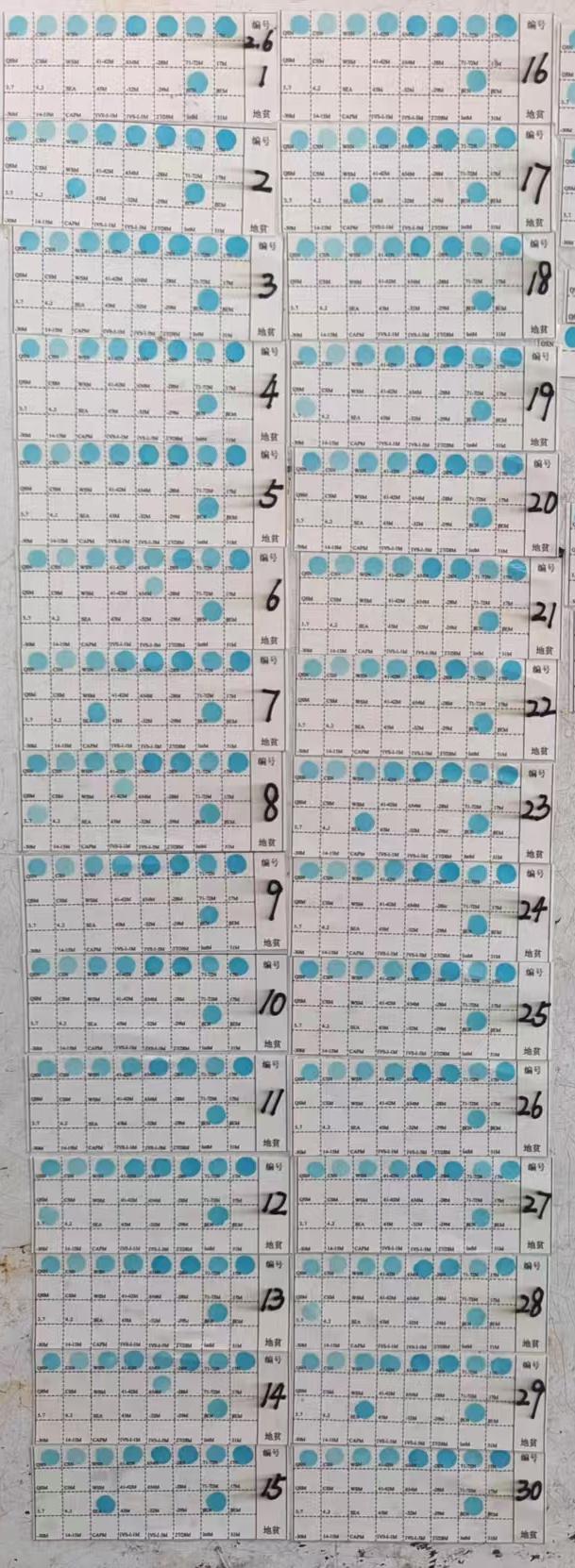

Supplement: S1 Fig — (ZIP) [file pone.0329365.s001.zip › S1_Fig13.jpg]

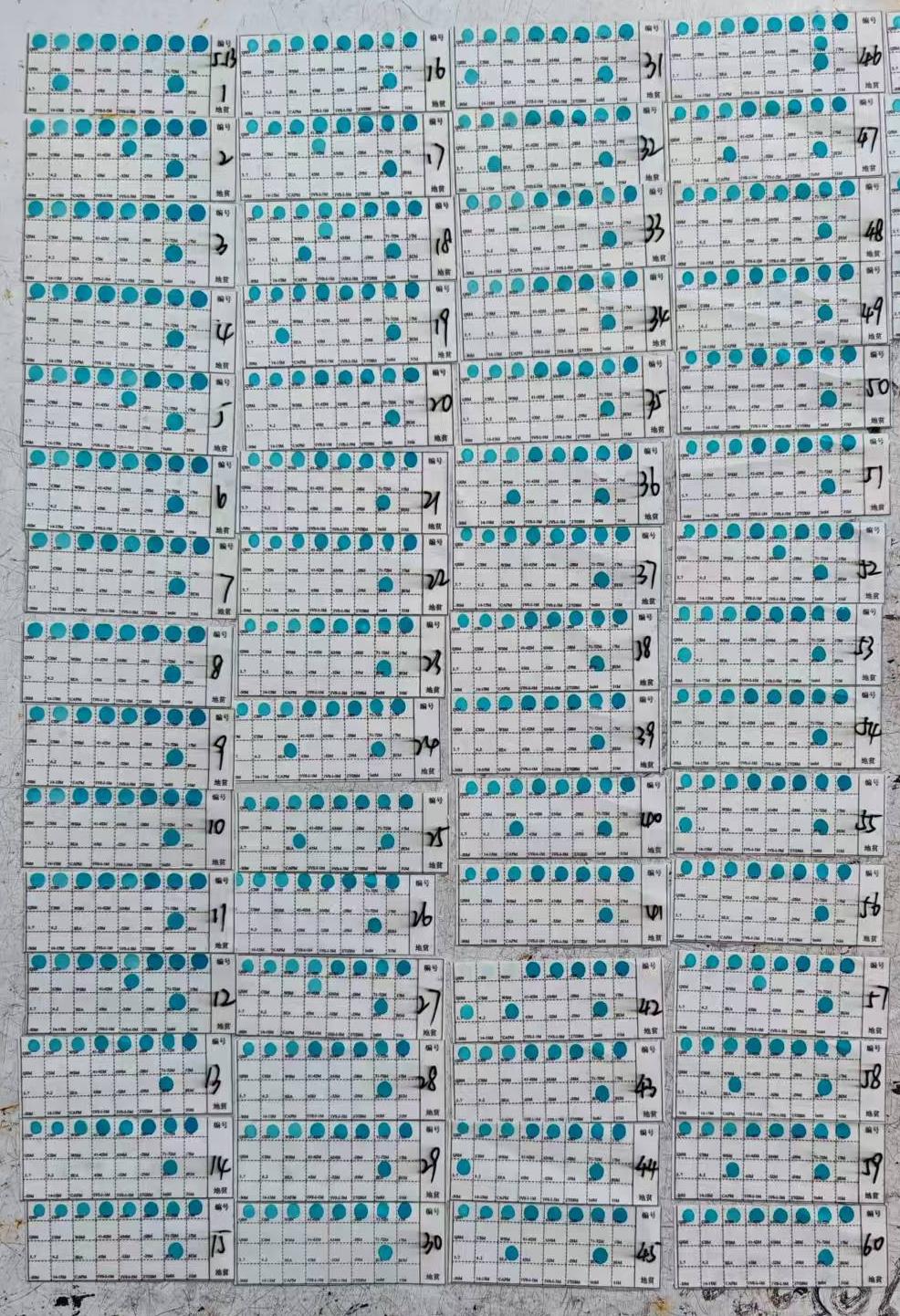

Supplement: S1 Fig — (ZIP) [file pone.0329365.s001.zip › S1_Fig14.jpg]

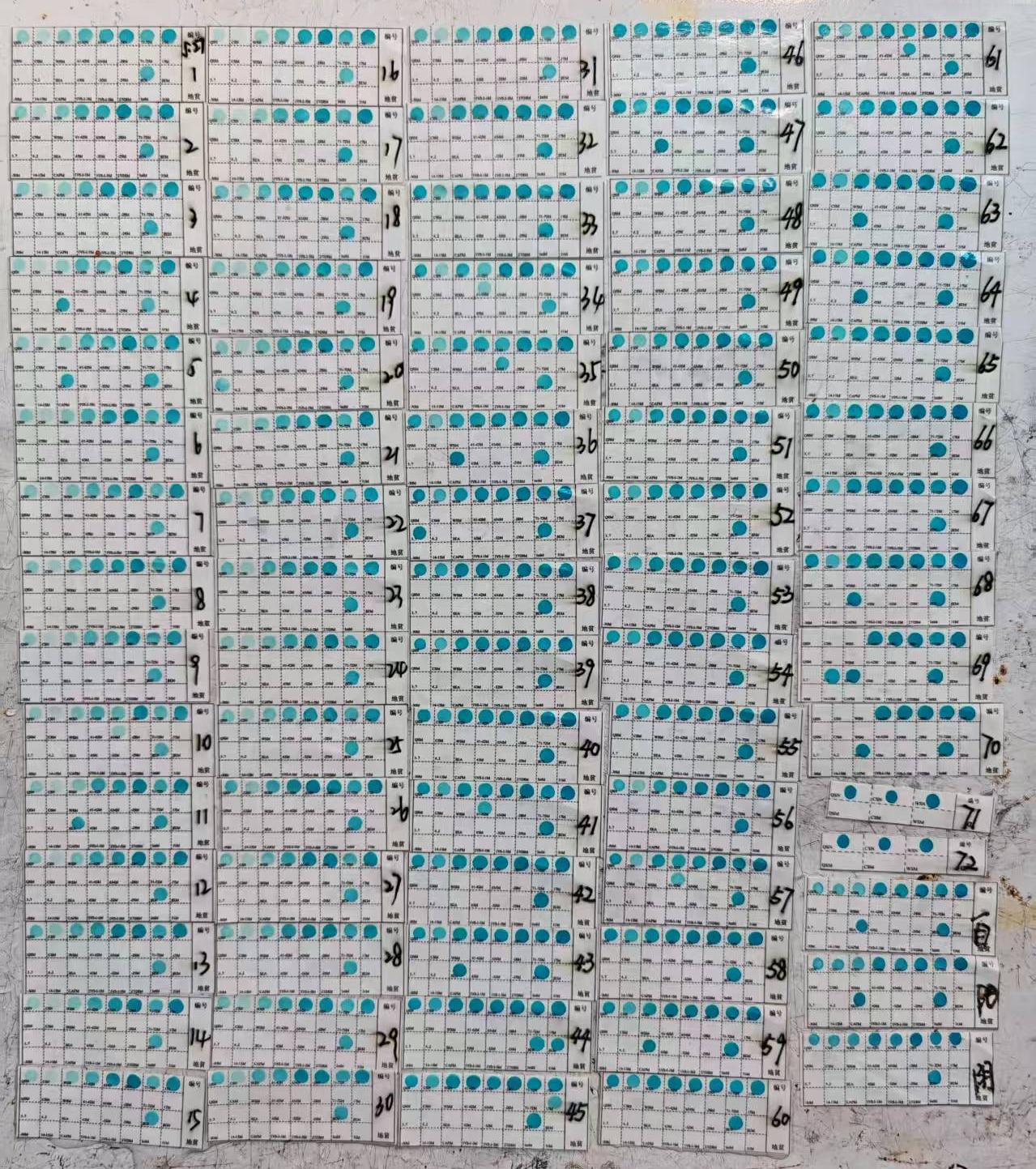

Supplement: S1 Fig — (ZIP) [file pone.0329365.s001.zip › S1_Fig15.jpg]

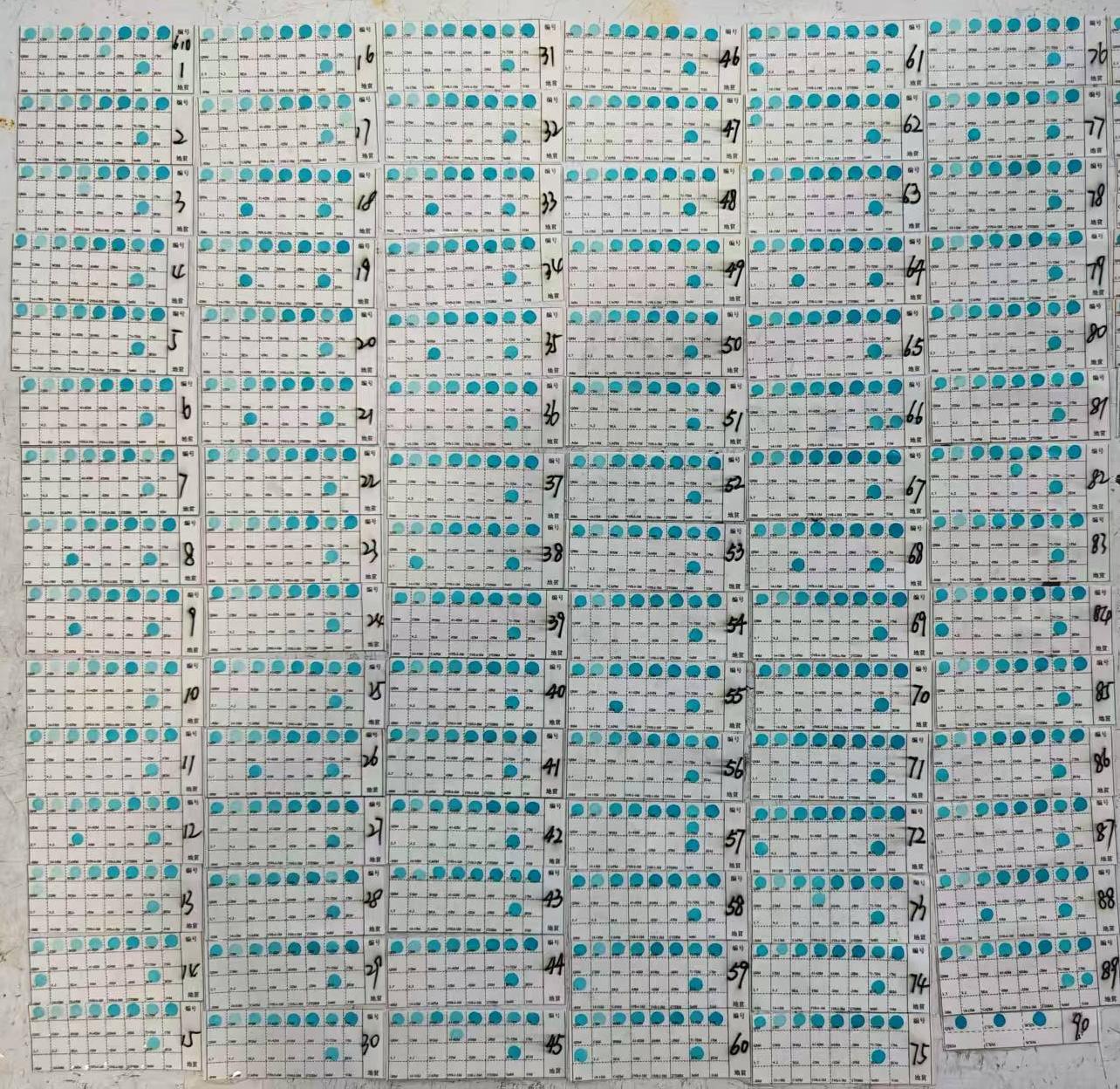

Supplement: S1 Fig — (ZIP) [file pone.0329365.s001.zip › S1_Fig16.jpg]

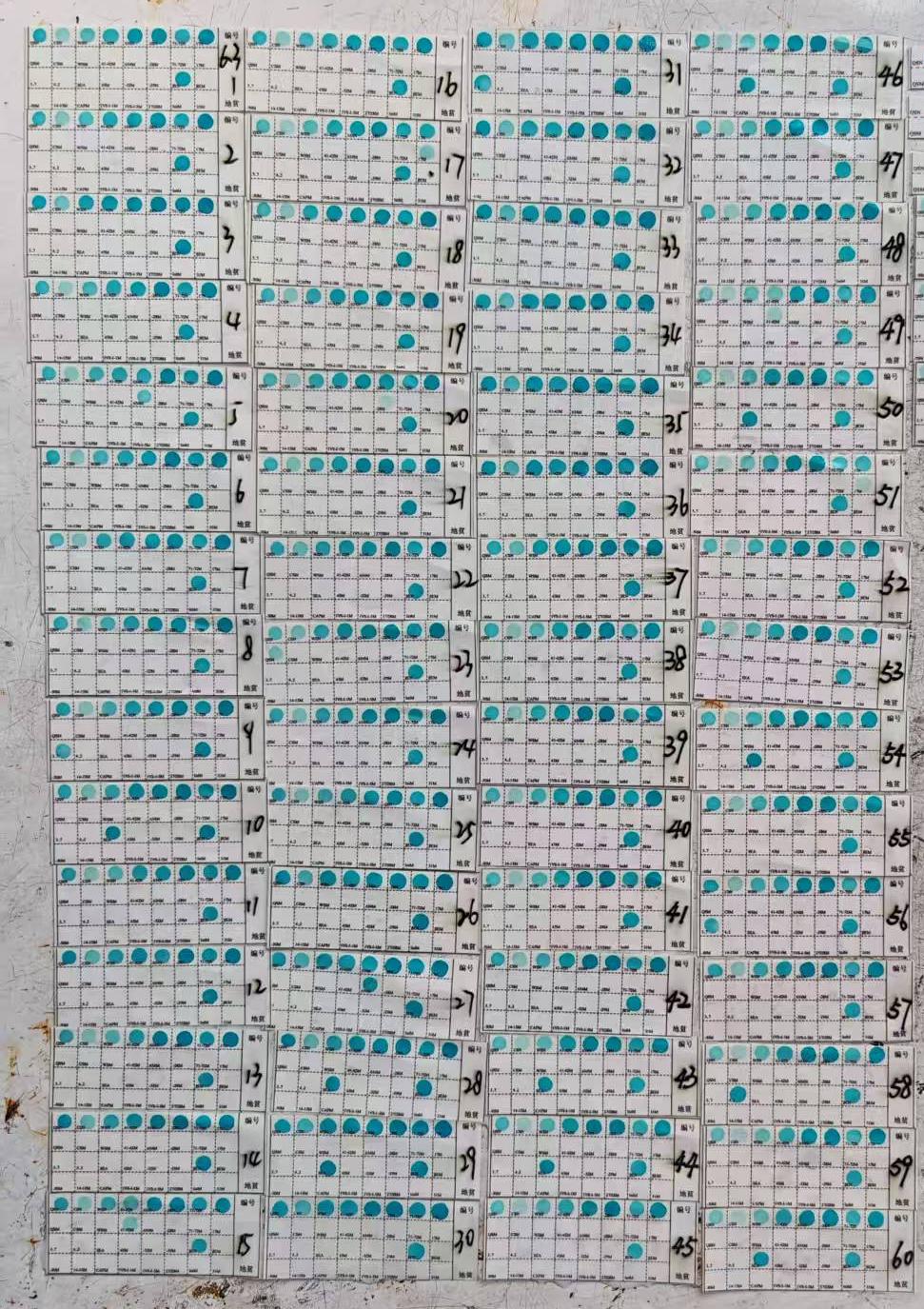

Supplement: S1 Fig — (ZIP) [file pone.0329365.s001.zip › S1_Fig17.jpg]

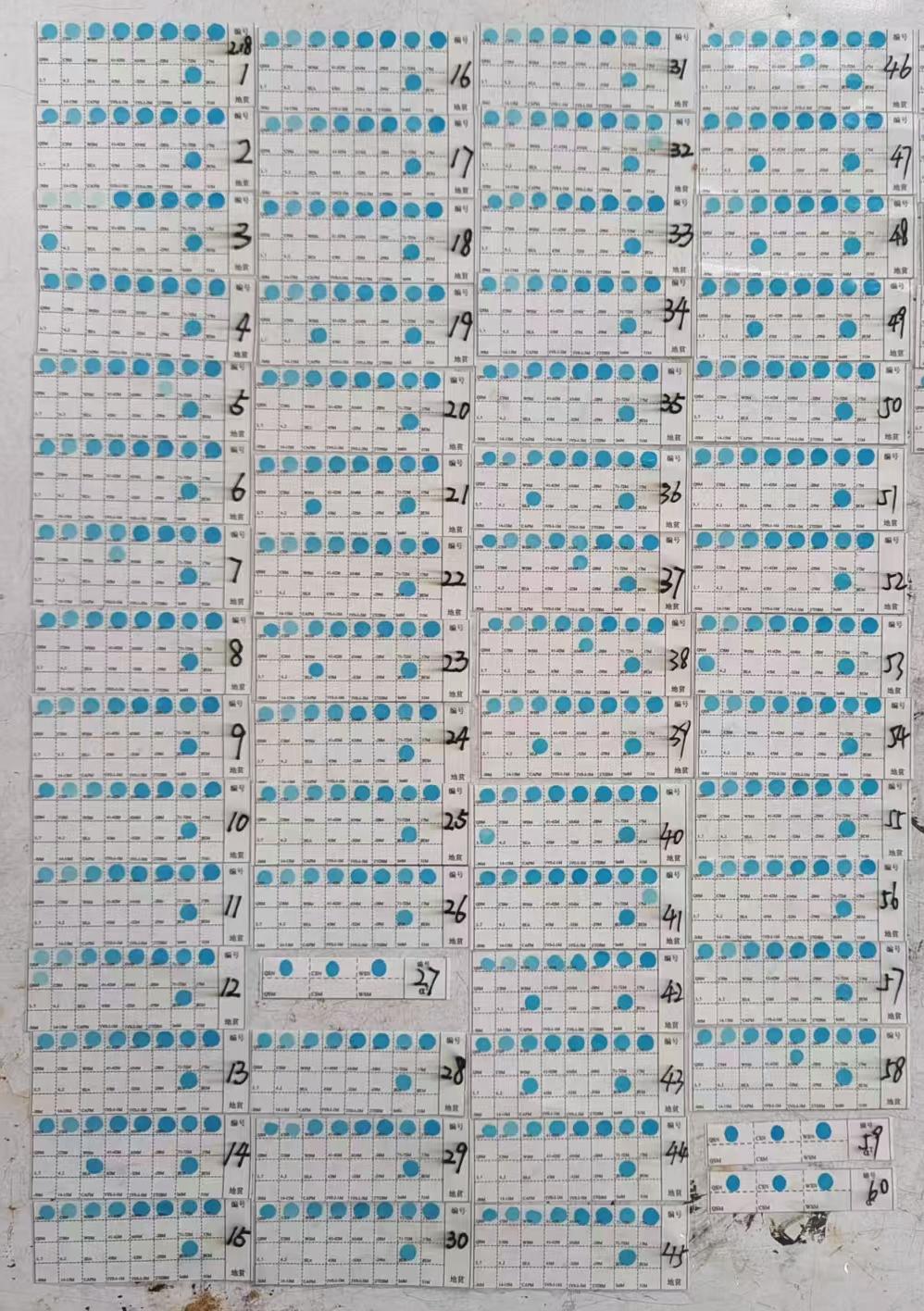

Supplement: S1 Fig — (ZIP) [file pone.0329365.s001.zip › S1_Fig18.jpg]

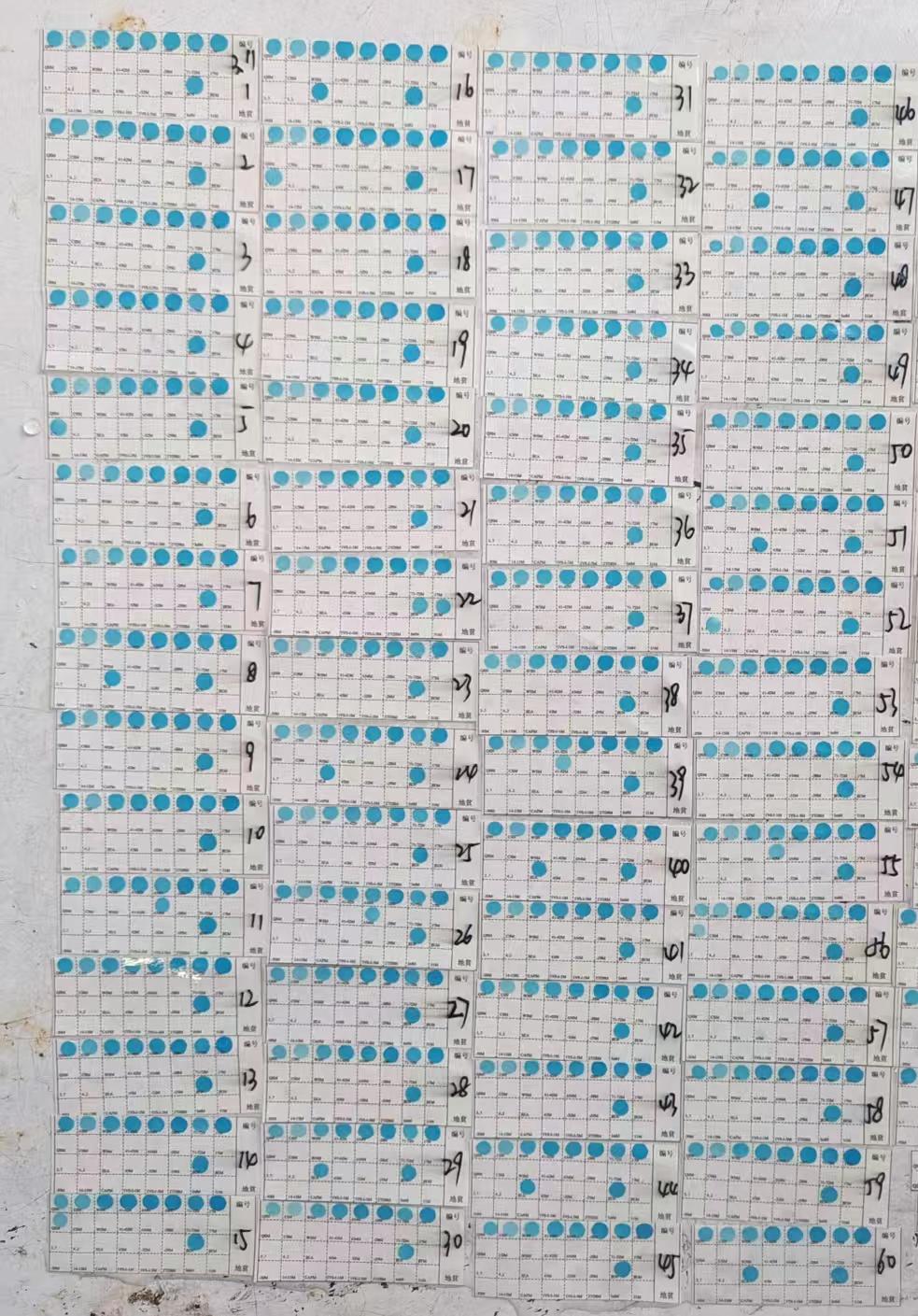

Supplement: S1 Fig — (ZIP) [file pone.0329365.s001.zip › S1_Fig19.jpg]

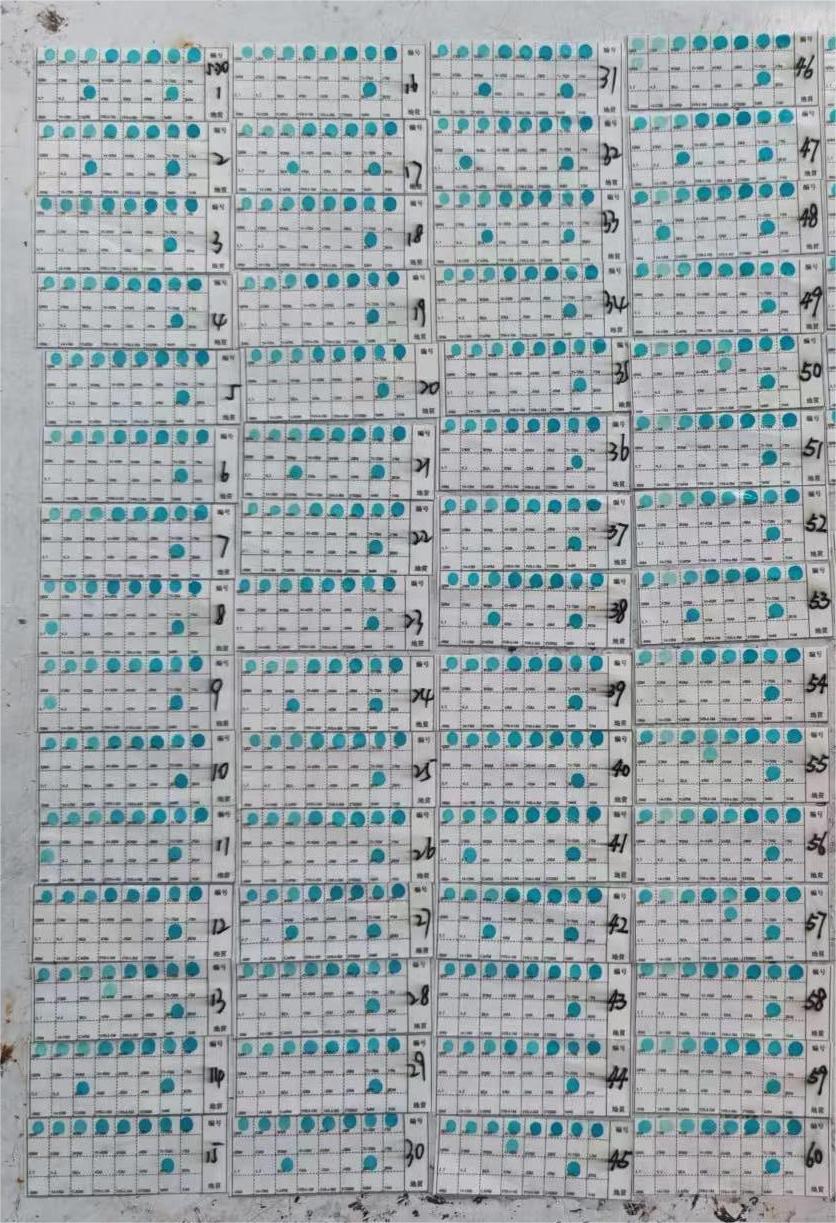

Supplement: S1 Fig — (ZIP) [file pone.0329365.s001.zip › S1_Fig2.jpg]

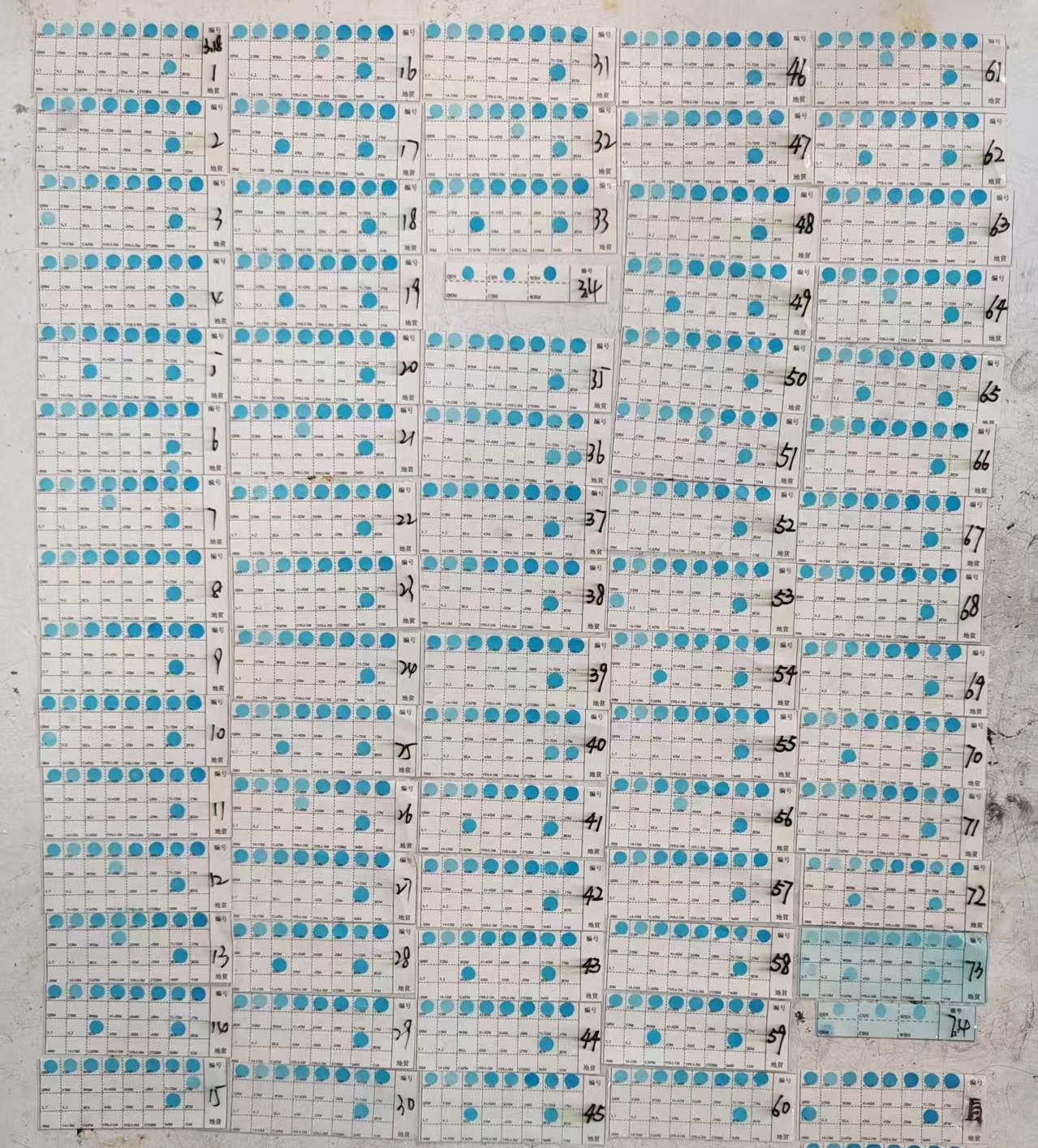

Supplement: S1 Fig — (ZIP) [file pone.0329365.s001.zip › S1_Fig20.jpg]

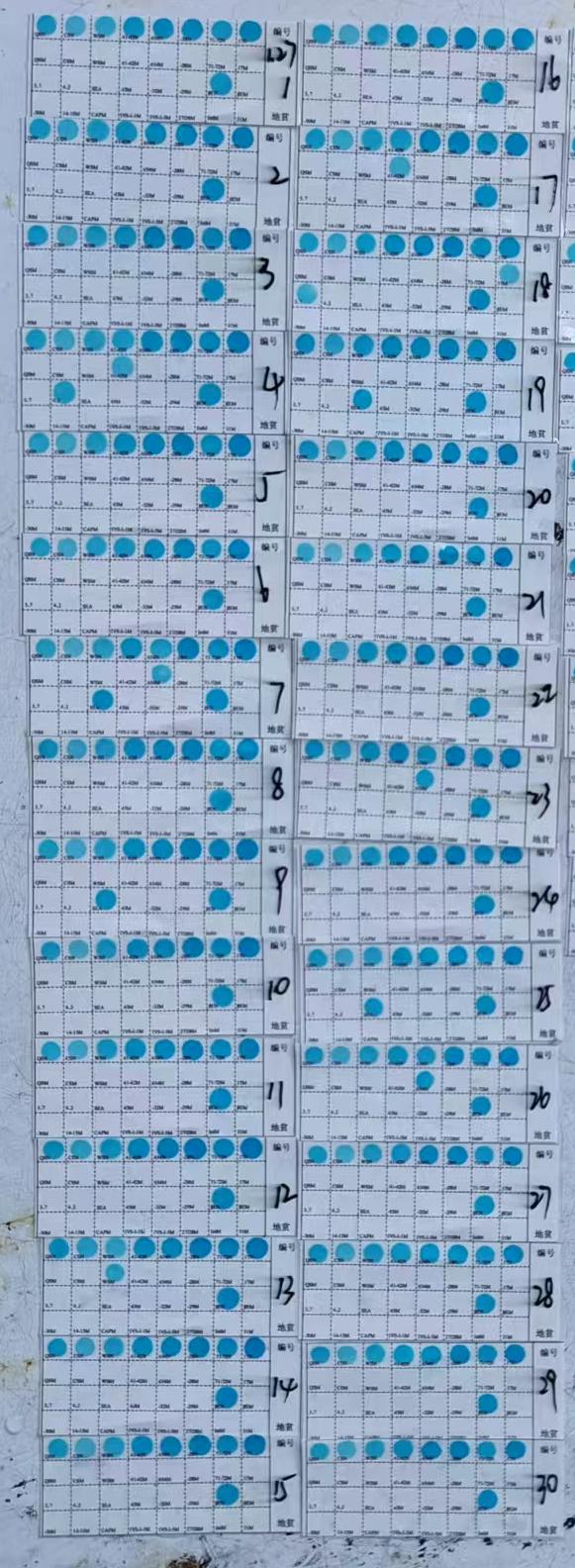

Supplement: S1 Fig — (ZIP) [file pone.0329365.s001.zip › S1_Fig3.jpg]

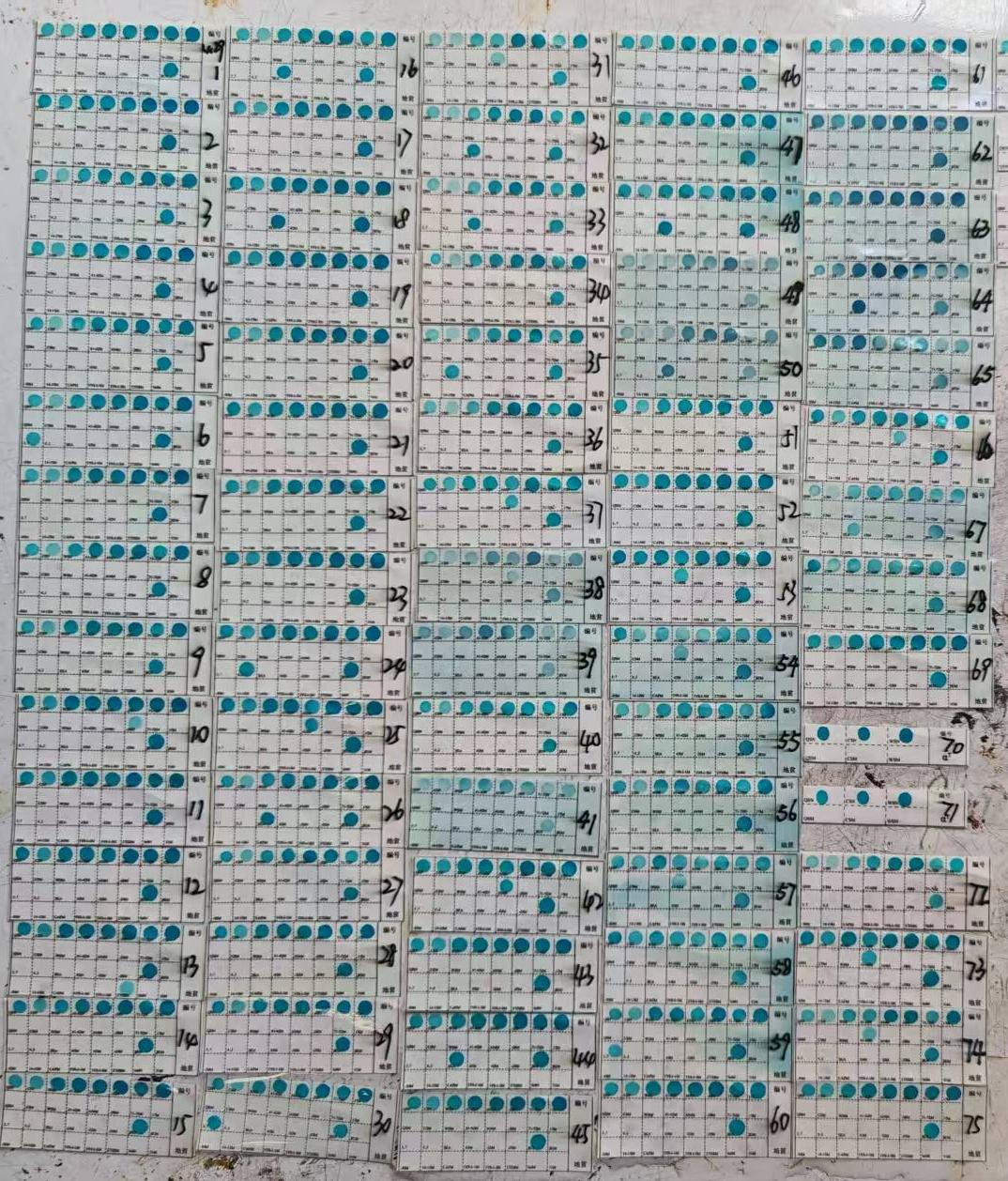

Supplement: S1 Fig — (ZIP) [file pone.0329365.s001.zip › S1_Fig4.jpg]

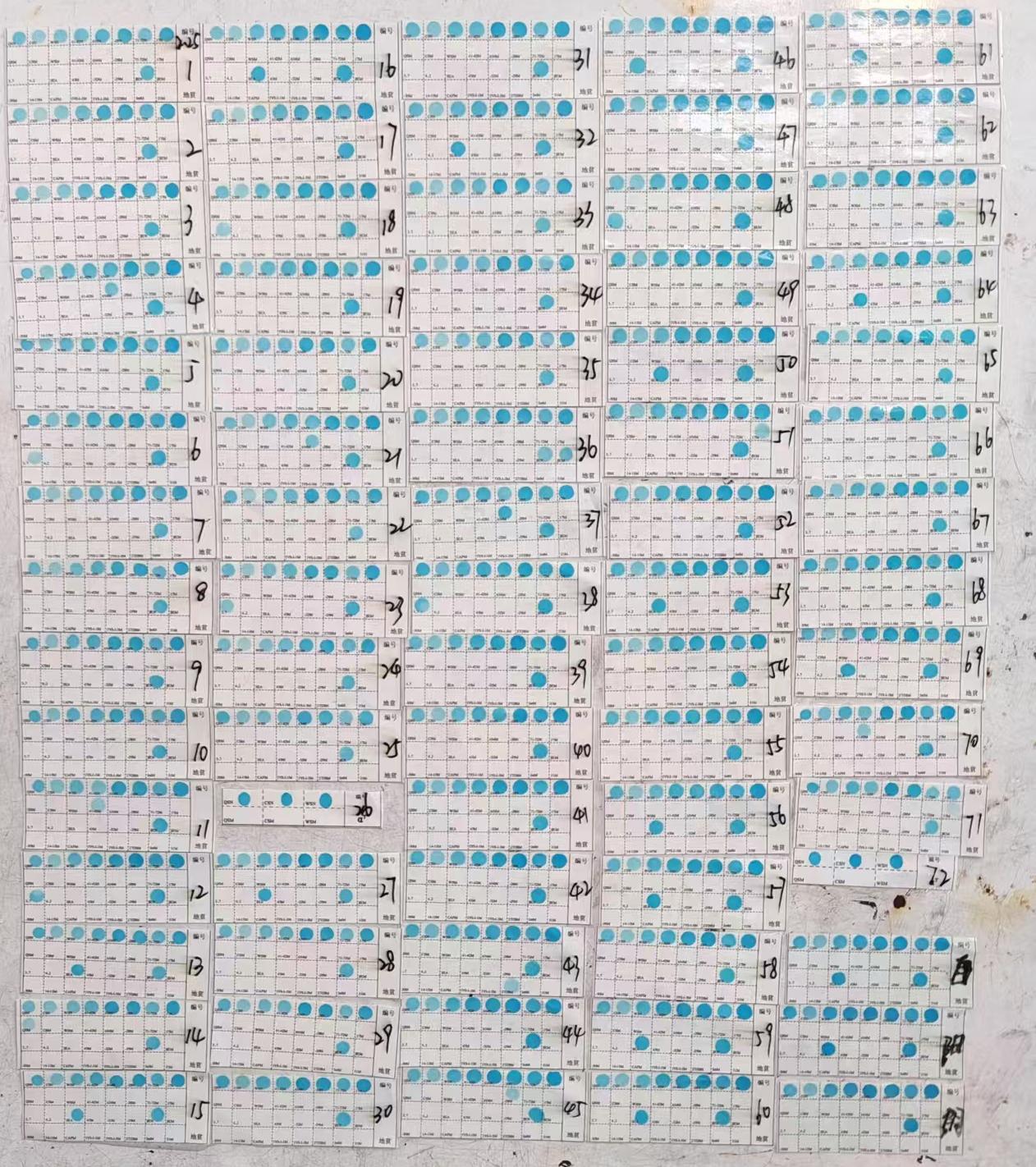

Supplement: S1 Fig — (ZIP) [file pone.0329365.s001.zip › S1_Fig5.jpg]

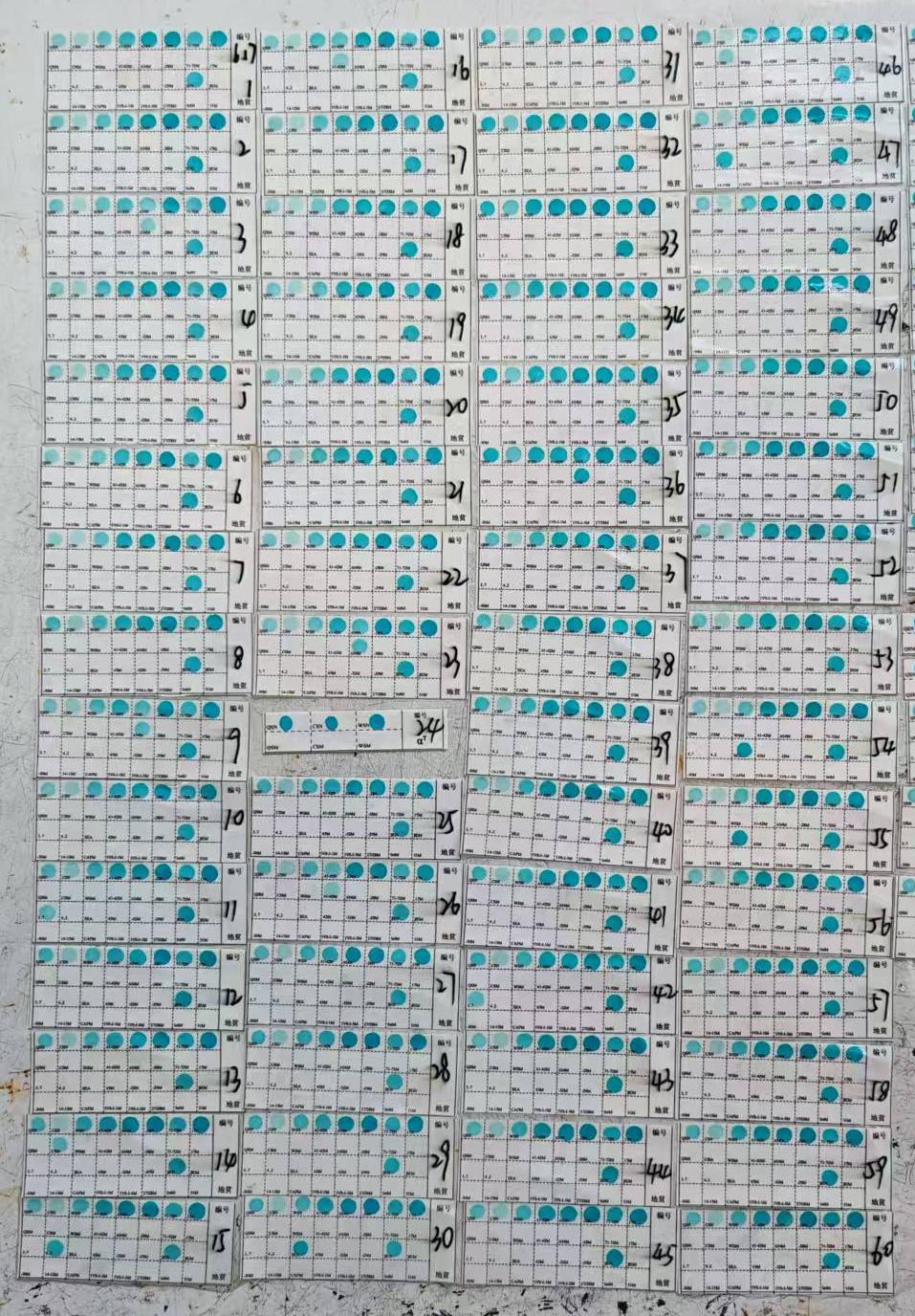

Supplement: S1 Fig — (ZIP) [file pone.0329365.s001.zip › S1_Fig6.jpg]

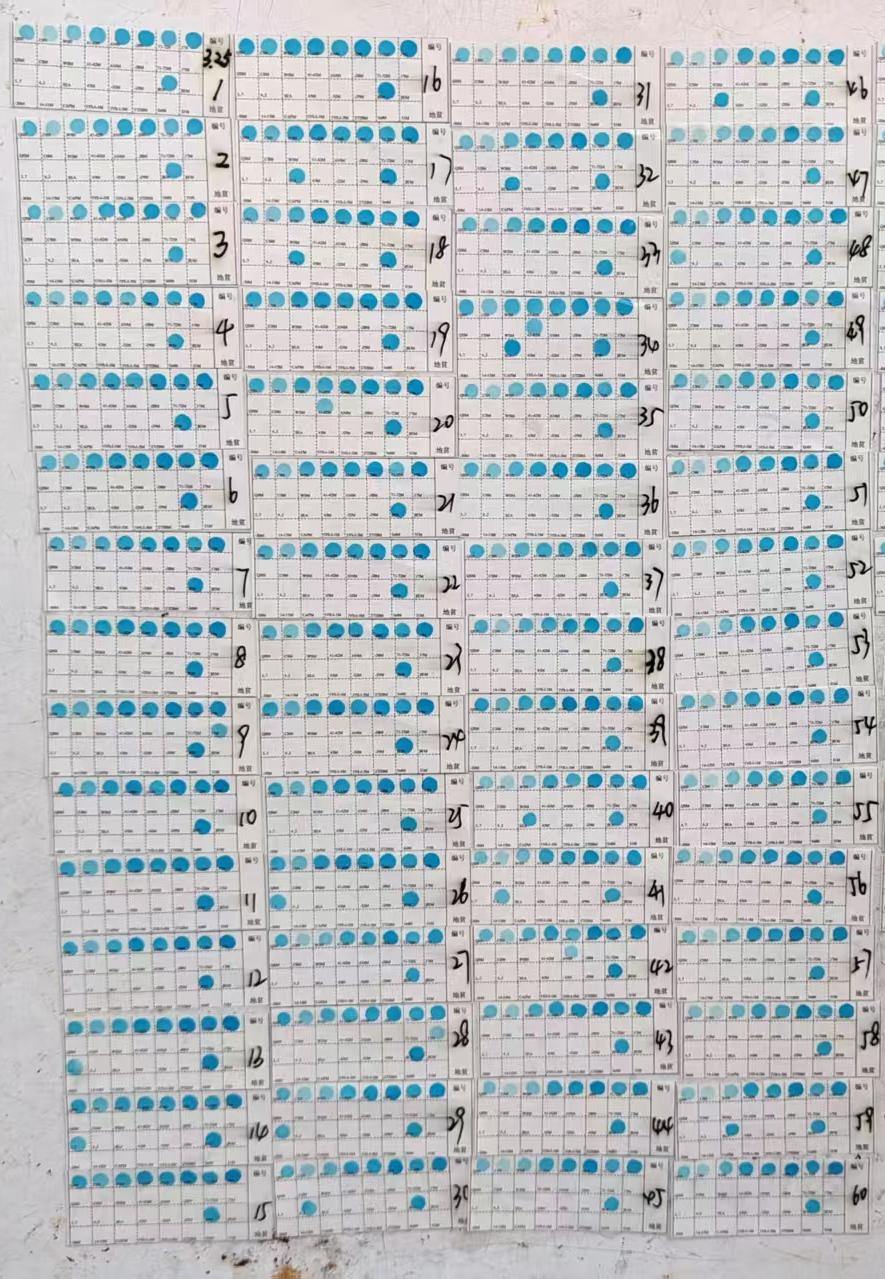

Supplement: S1 Fig — (ZIP) [file pone.0329365.s001.zip › S1_Fig7.jpg]

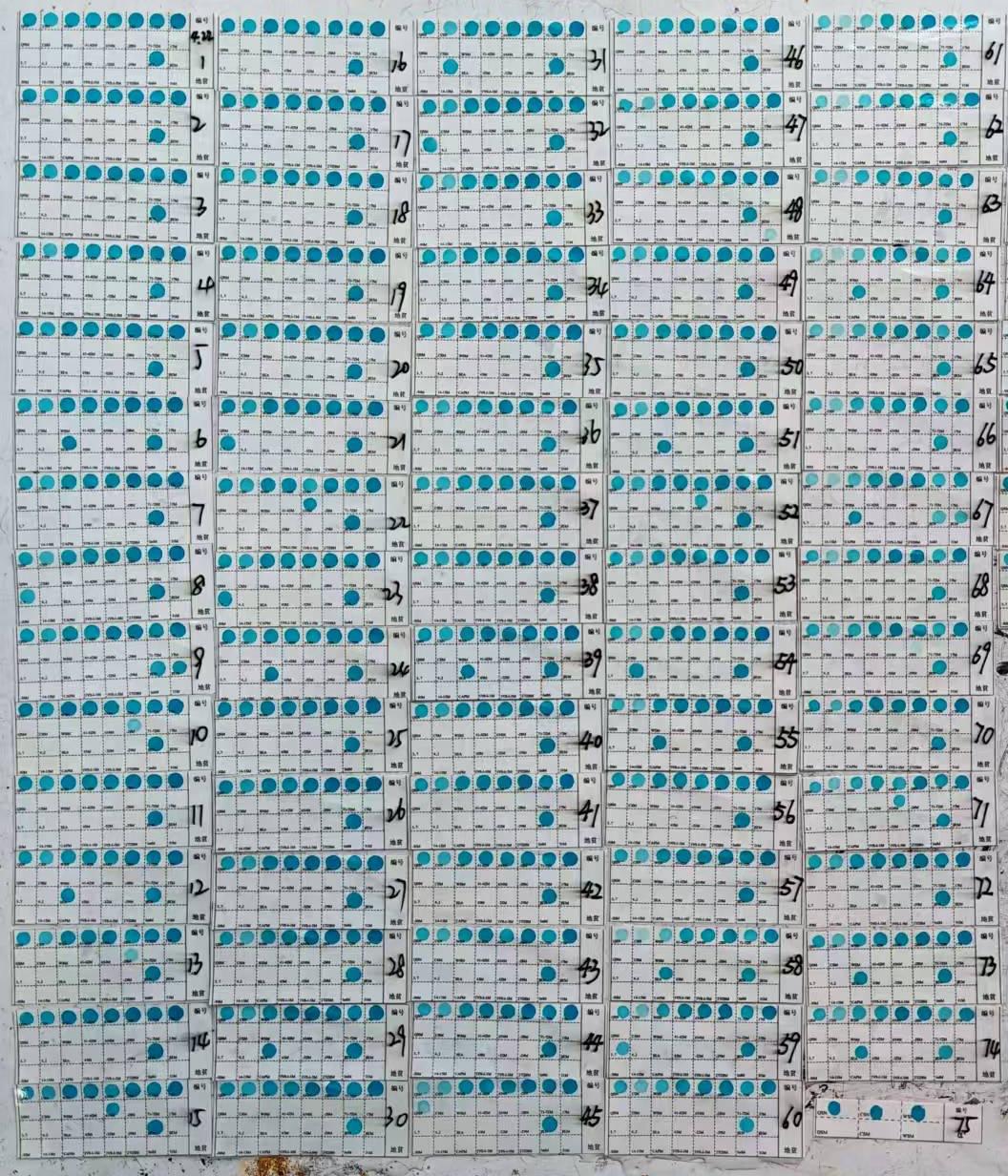

Supplement: S1 Fig — (ZIP) [file pone.0329365.s001.zip › S1_Fig8.jpg]

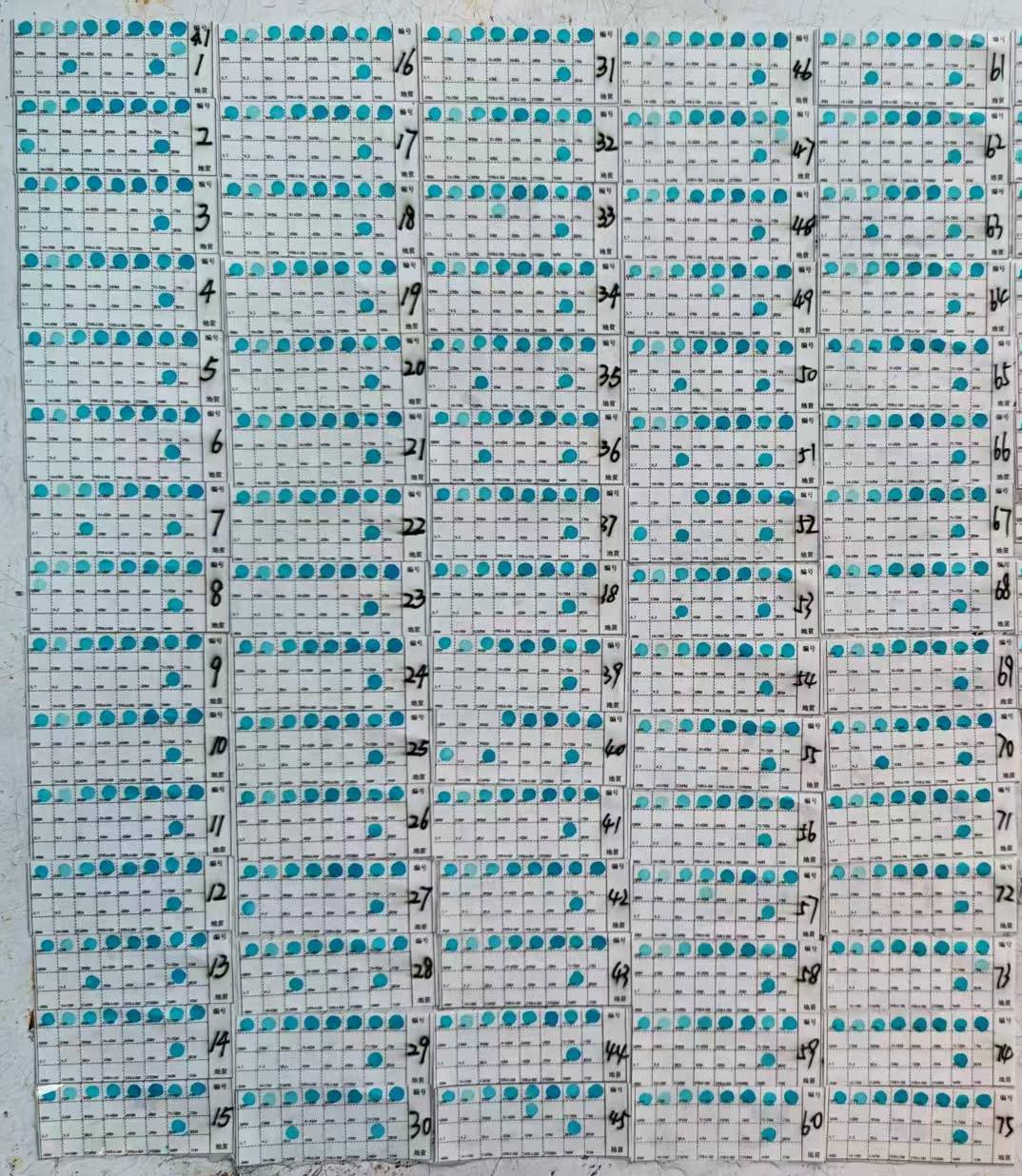

Supplement: S1 Fig — (ZIP) [file pone.0329365.s001.zip › S1_Fig9.jpg]

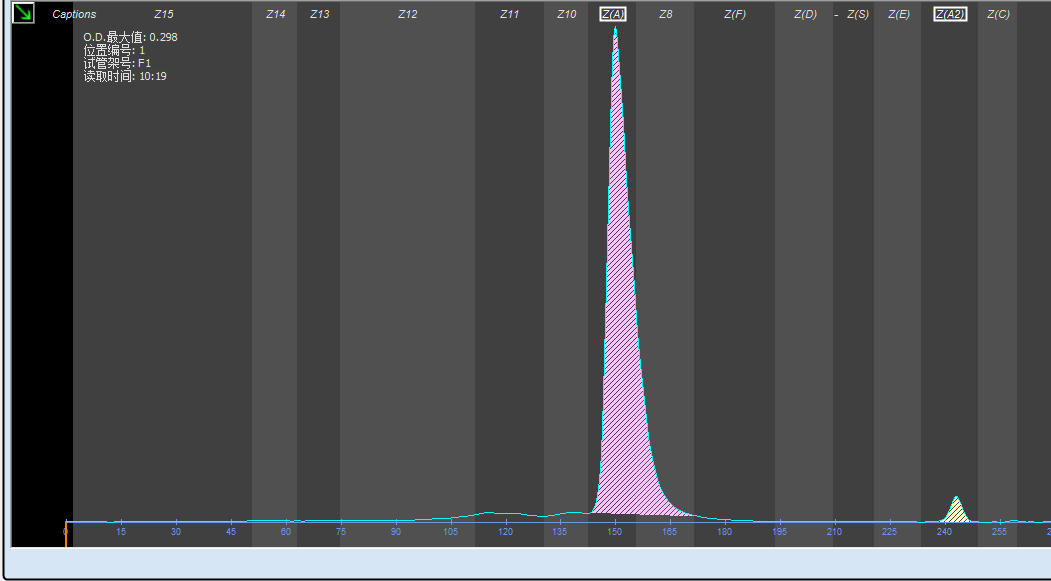

Supplement: S2 Fig — (ZIP) [file pone.0329365.s002.zip › S2_Fig1.png]

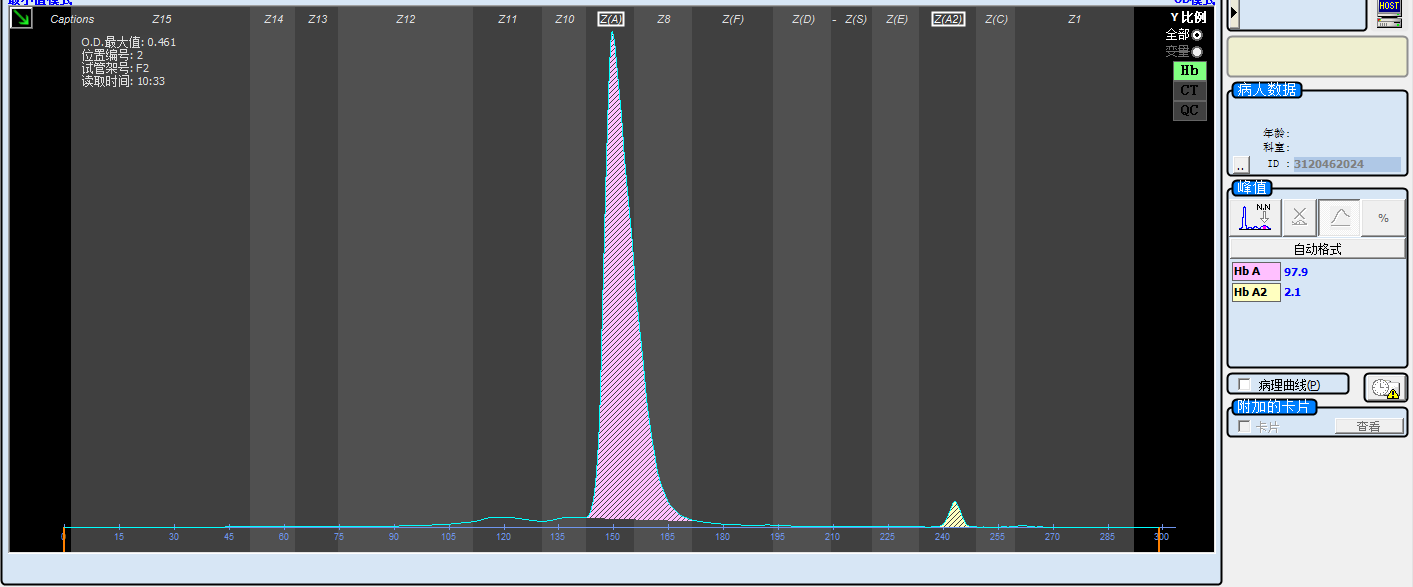

Supplement: S2 Fig — (ZIP) [file pone.0329365.s002.zip › S2_Fig10.png]

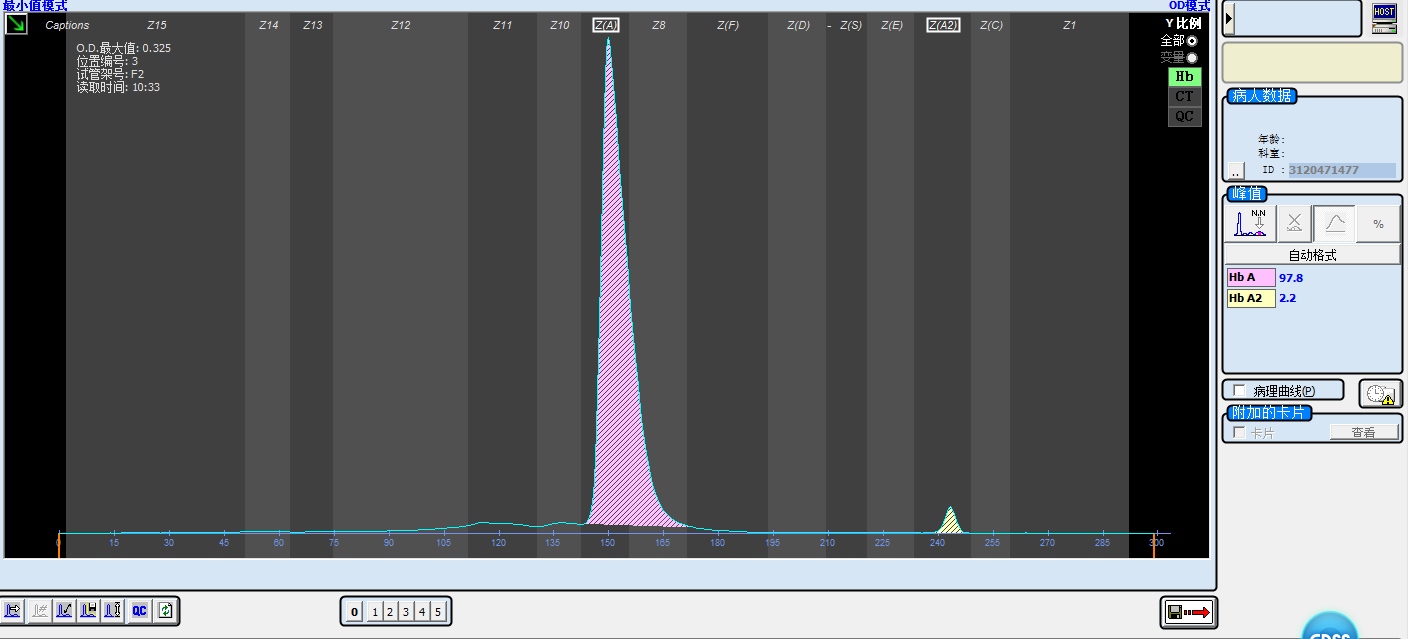

Supplement: S2 Fig — (ZIP) [file pone.0329365.s002.zip › S2_Fig11.png]

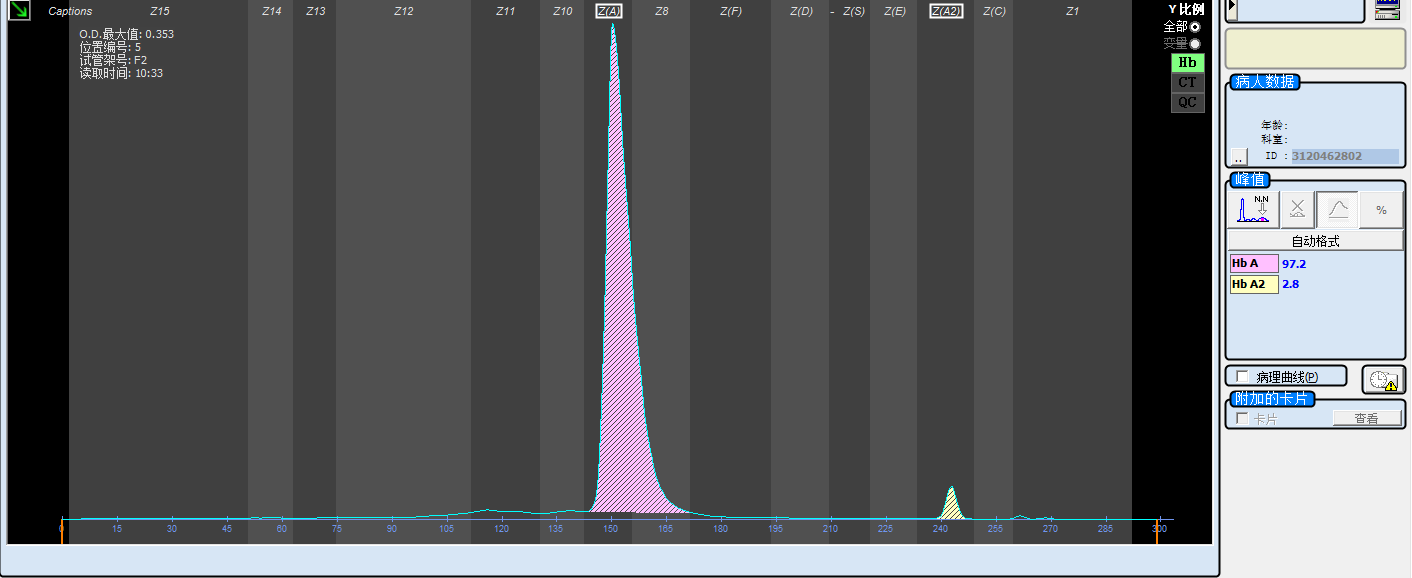

Supplement: S2 Fig — (ZIP) [file pone.0329365.s002.zip › S2_Fig12.png]

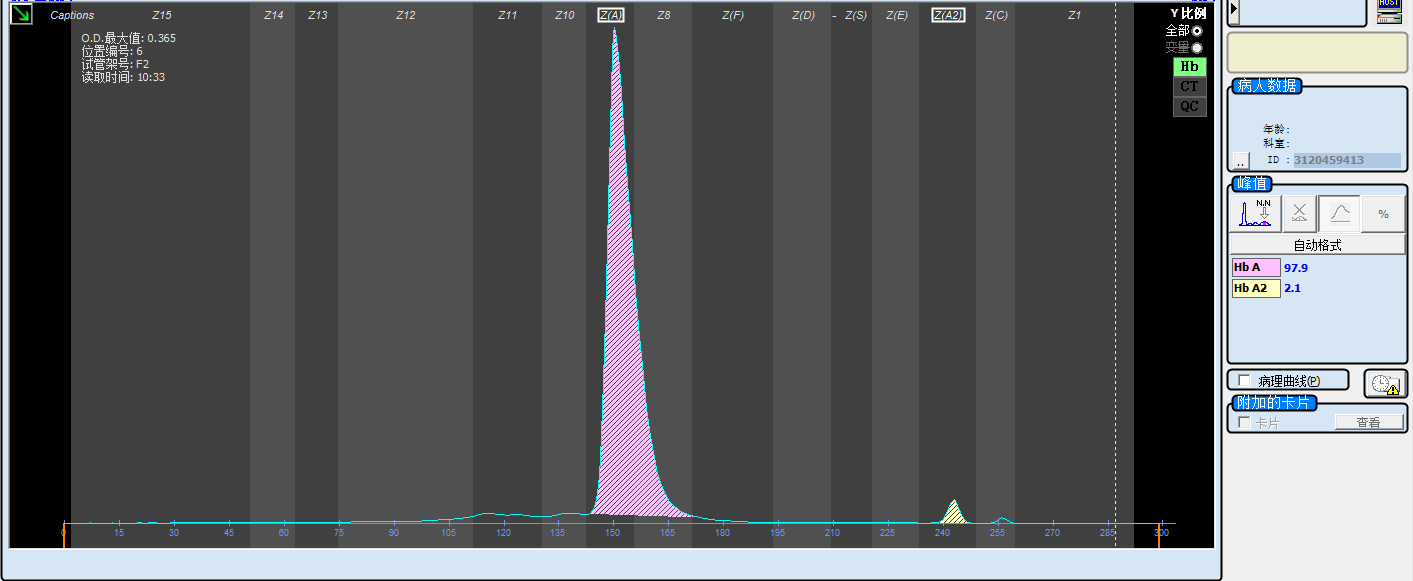

Supplement: S2 Fig — (ZIP) [file pone.0329365.s002.zip › S2_Fig13.png]

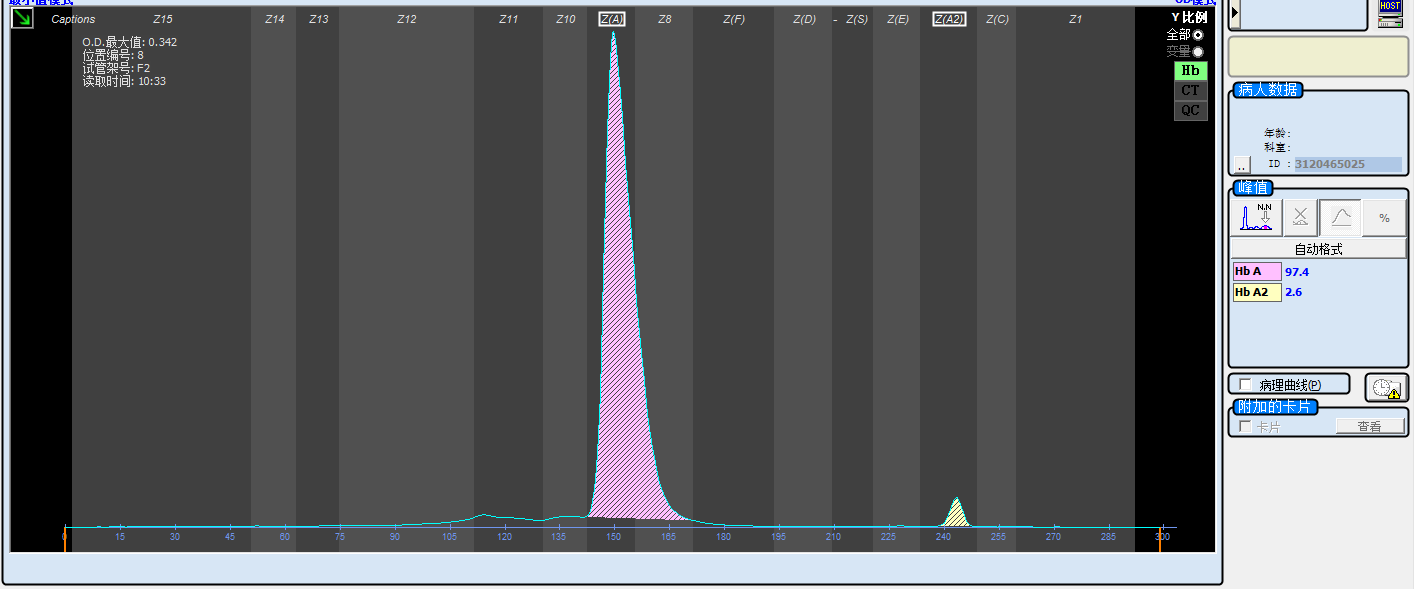

Supplement: S2 Fig — (ZIP) [file pone.0329365.s002.zip › S2_Fig14.png]

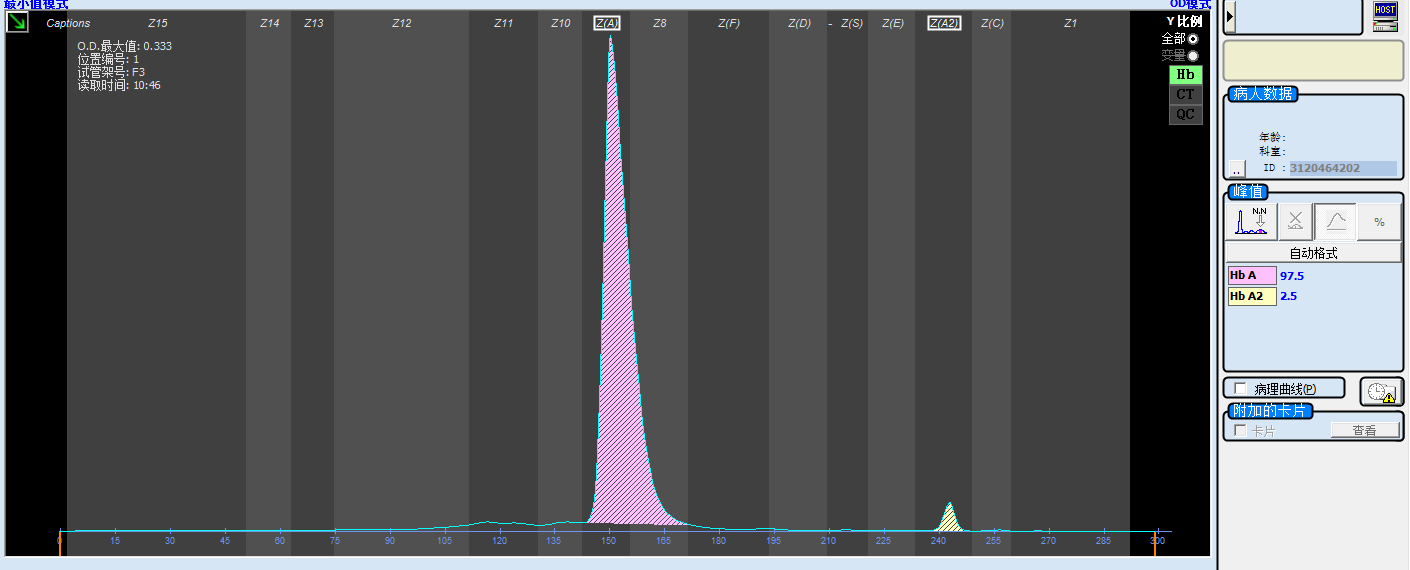

Supplement: S2 Fig — (ZIP) [file pone.0329365.s002.zip › S2_Fig15.png]

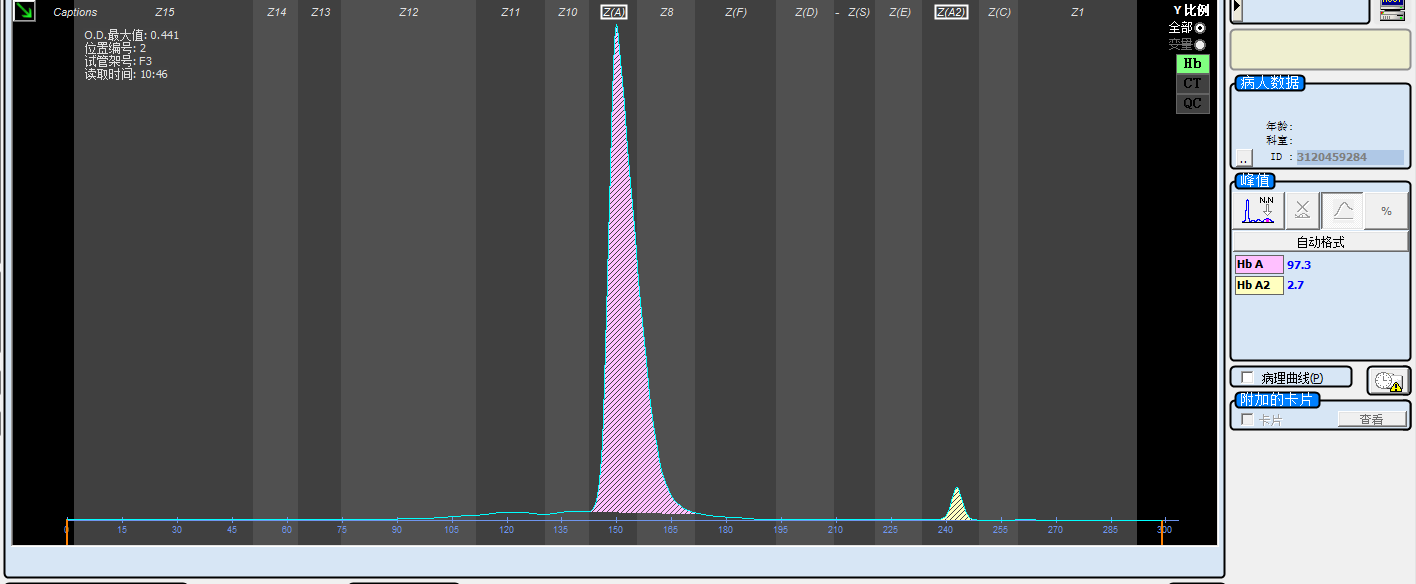

Supplement: S2 Fig — (ZIP) [file pone.0329365.s002.zip › S2_Fig16.png]

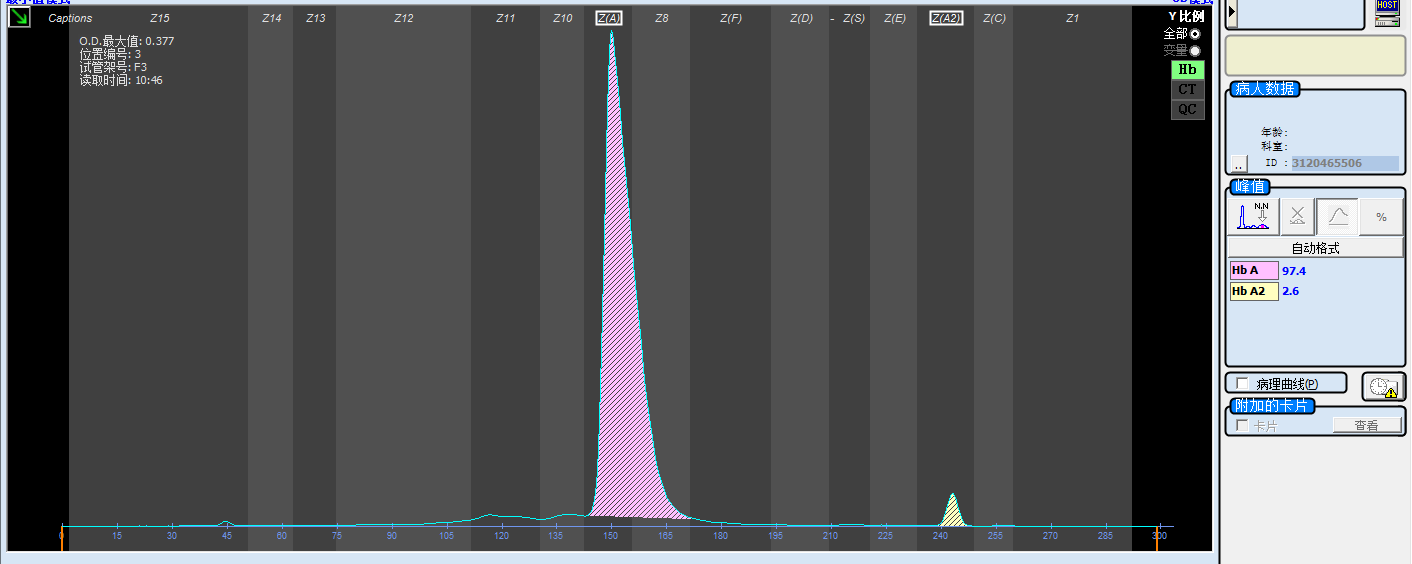

Supplement: S2 Fig — (ZIP) [file pone.0329365.s002.zip › S2_Fig17.png]

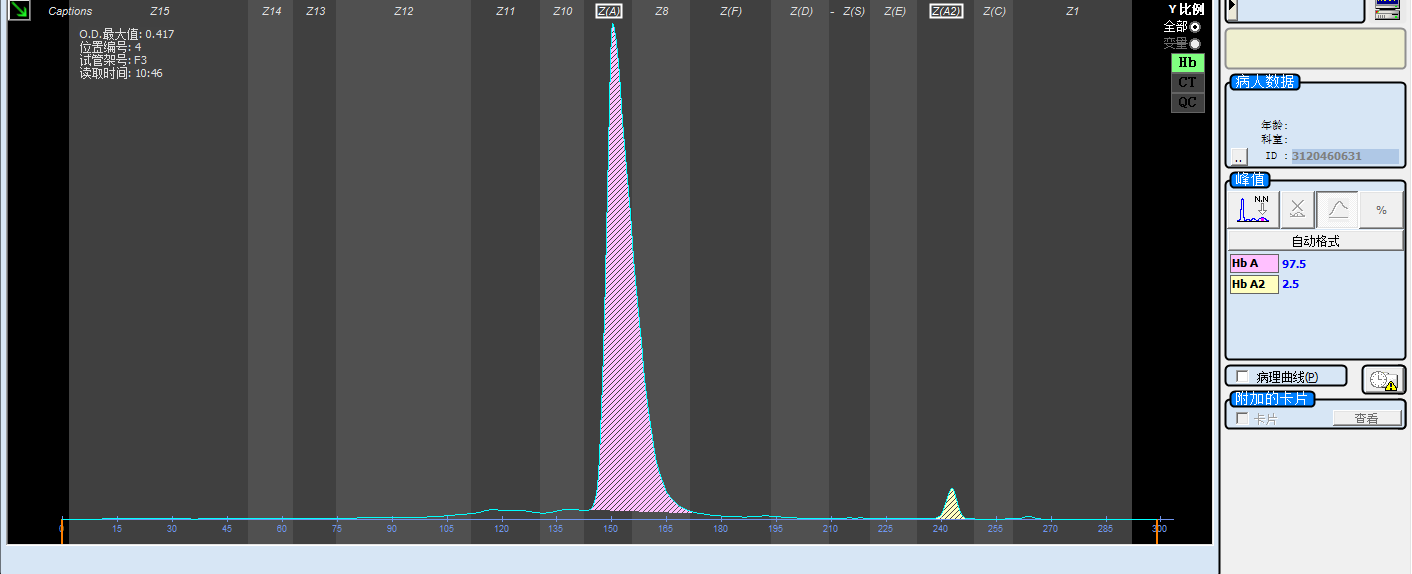

Supplement: S2 Fig — (ZIP) [file pone.0329365.s002.zip › S2_Fig18.png]

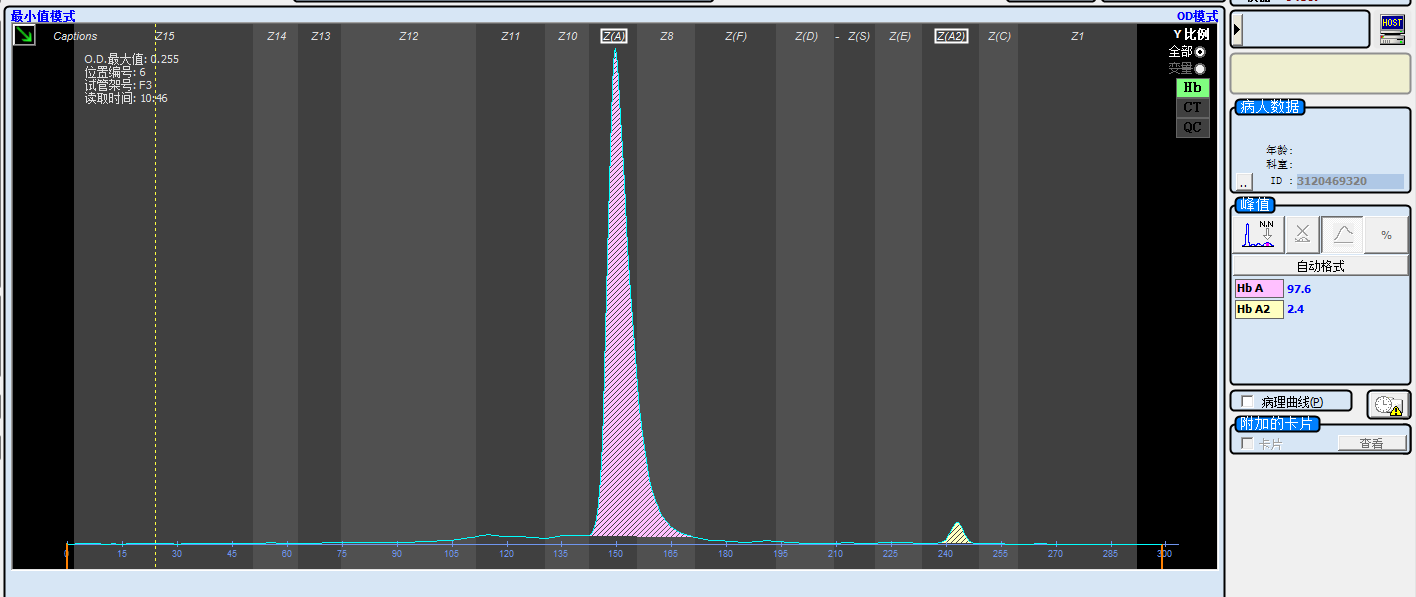

Supplement: S2 Fig — (ZIP) [file pone.0329365.s002.zip › S2_Fig19.png]

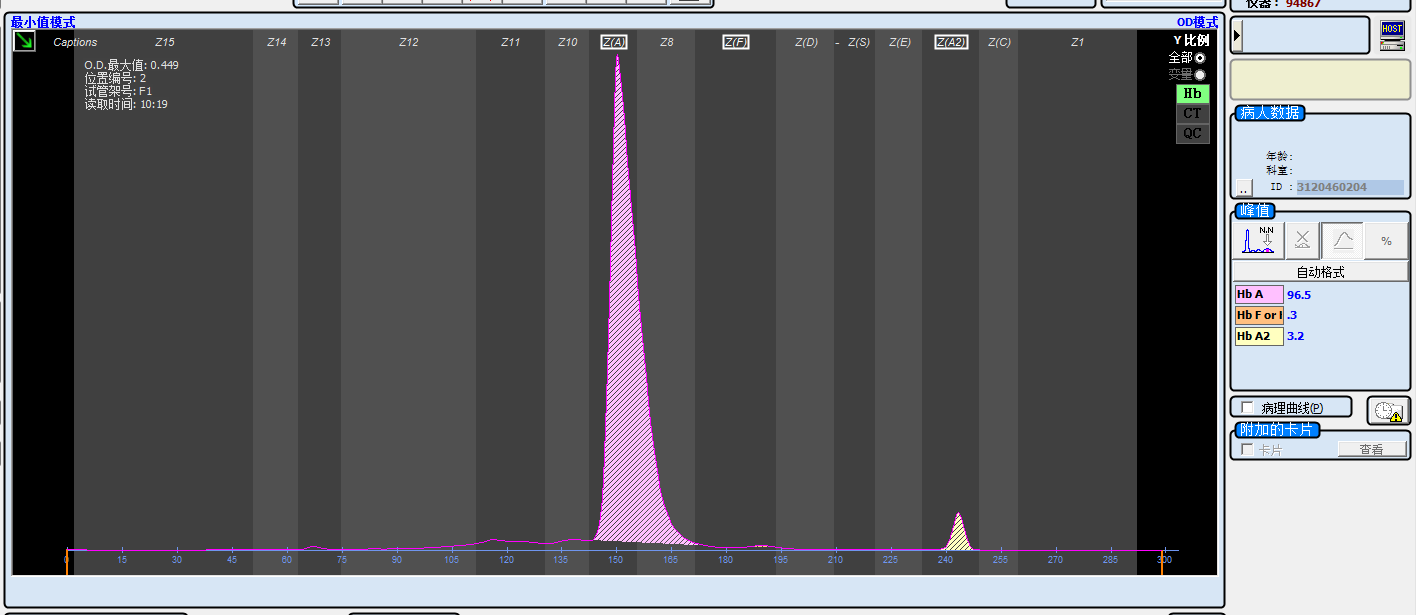

Supplement: S2 Fig — (ZIP) [file pone.0329365.s002.zip › S2_Fig2.png]

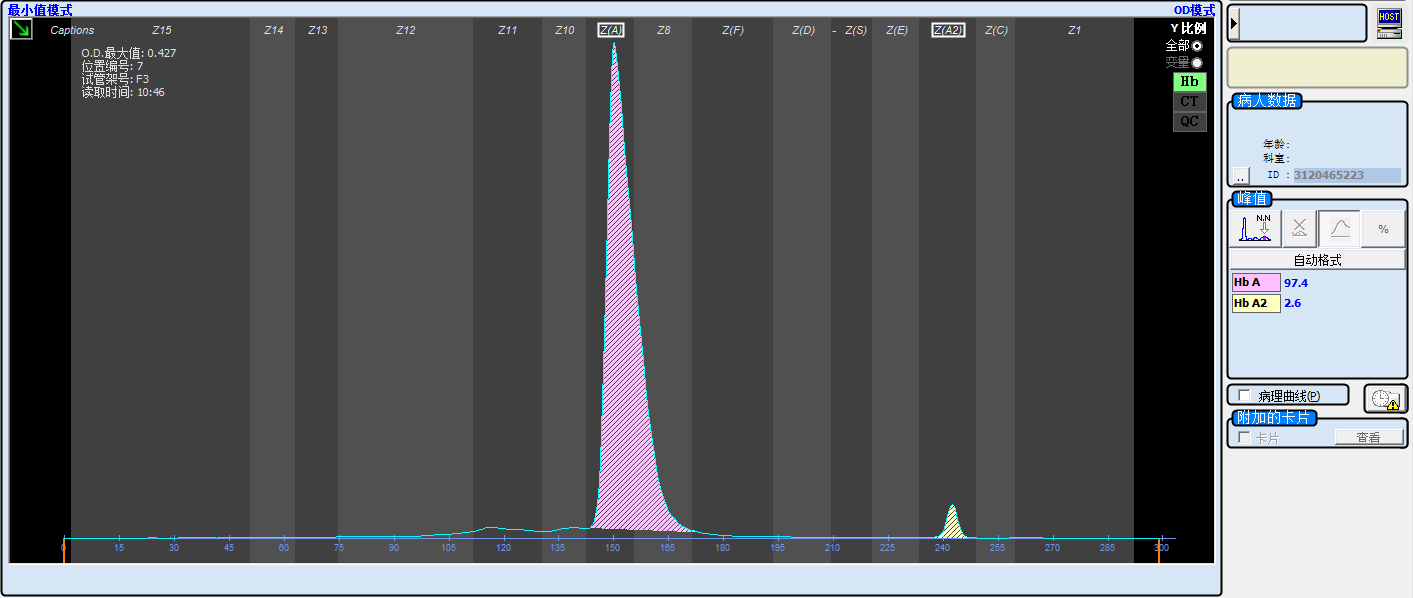

Supplement: S2 Fig — (ZIP) [file pone.0329365.s002.zip › S2_Fig20.png]

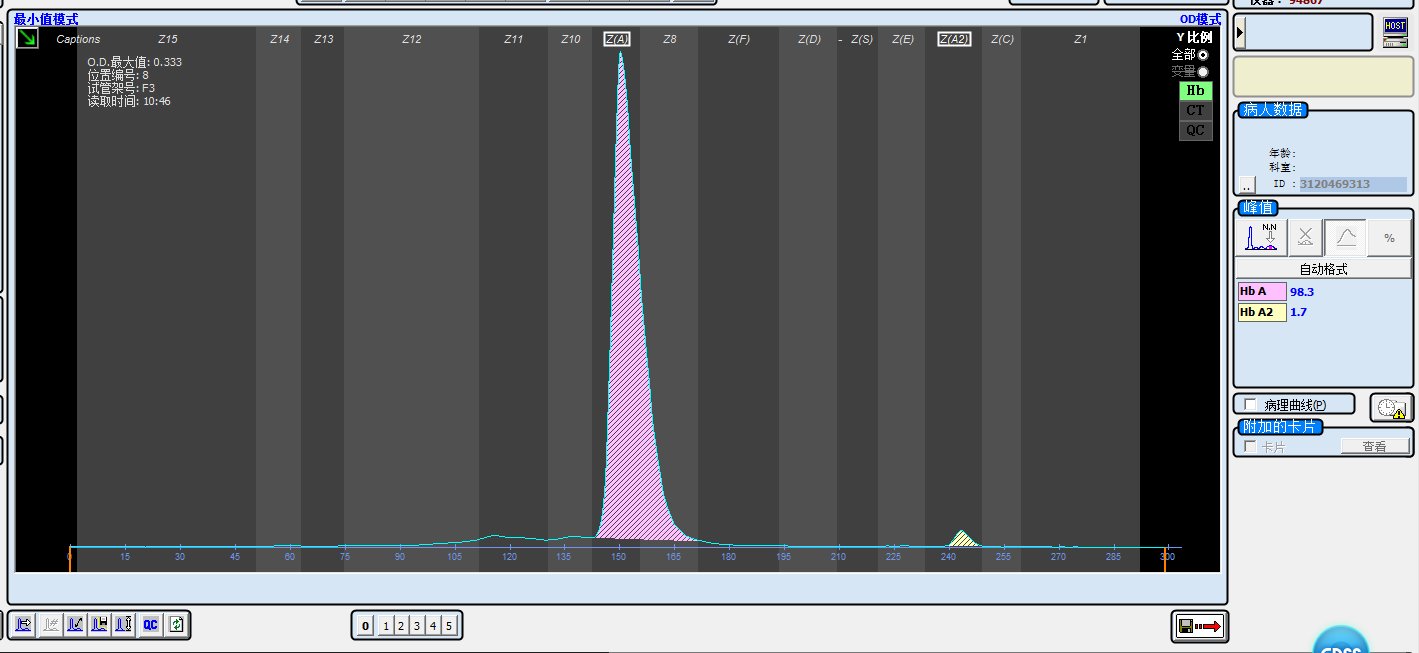

Supplement: S2 Fig — (ZIP) [file pone.0329365.s002.zip › S2_Fig21.png]

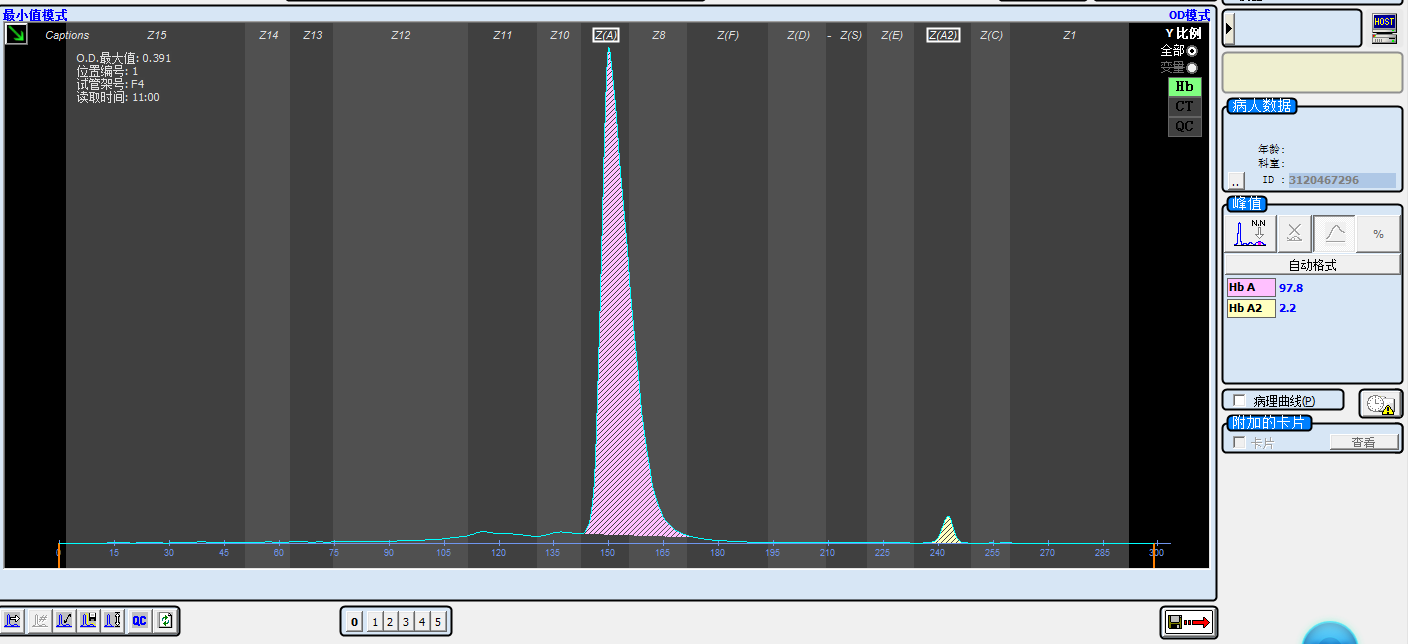

Supplement: S2 Fig — (ZIP) [file pone.0329365.s002.zip › S2_Fig22.png]

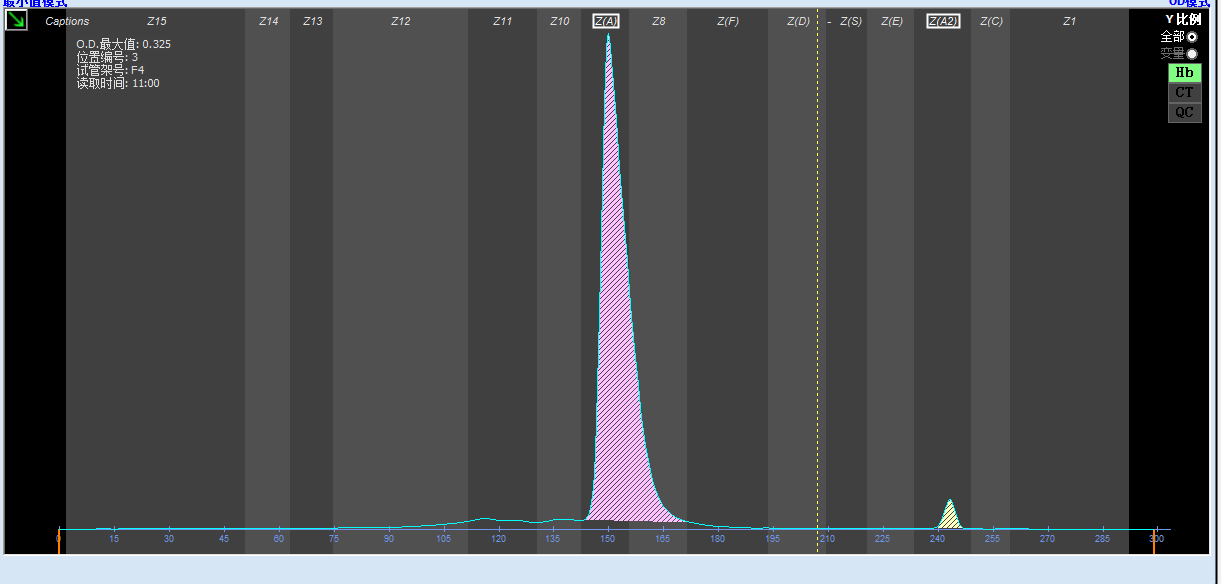

Supplement: S2 Fig — (ZIP) [file pone.0329365.s002.zip › S2_Fig23.png]

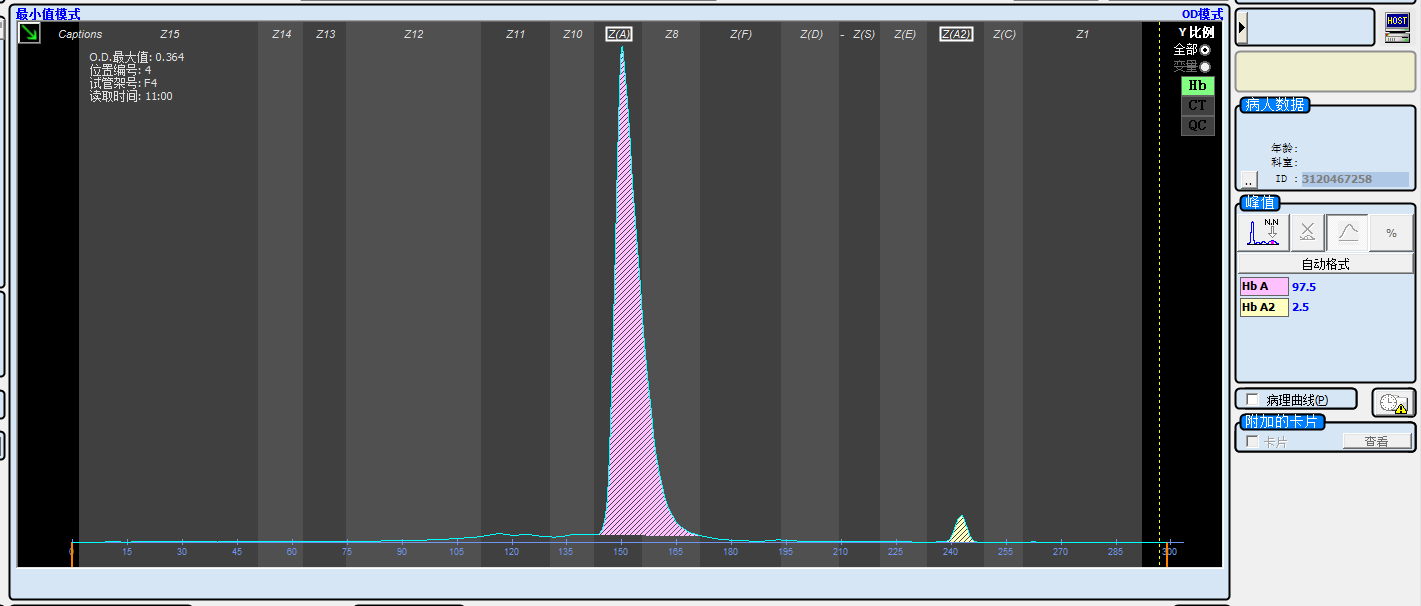

Supplement: S2 Fig — (ZIP) [file pone.0329365.s002.zip › S2_Fig24.png]

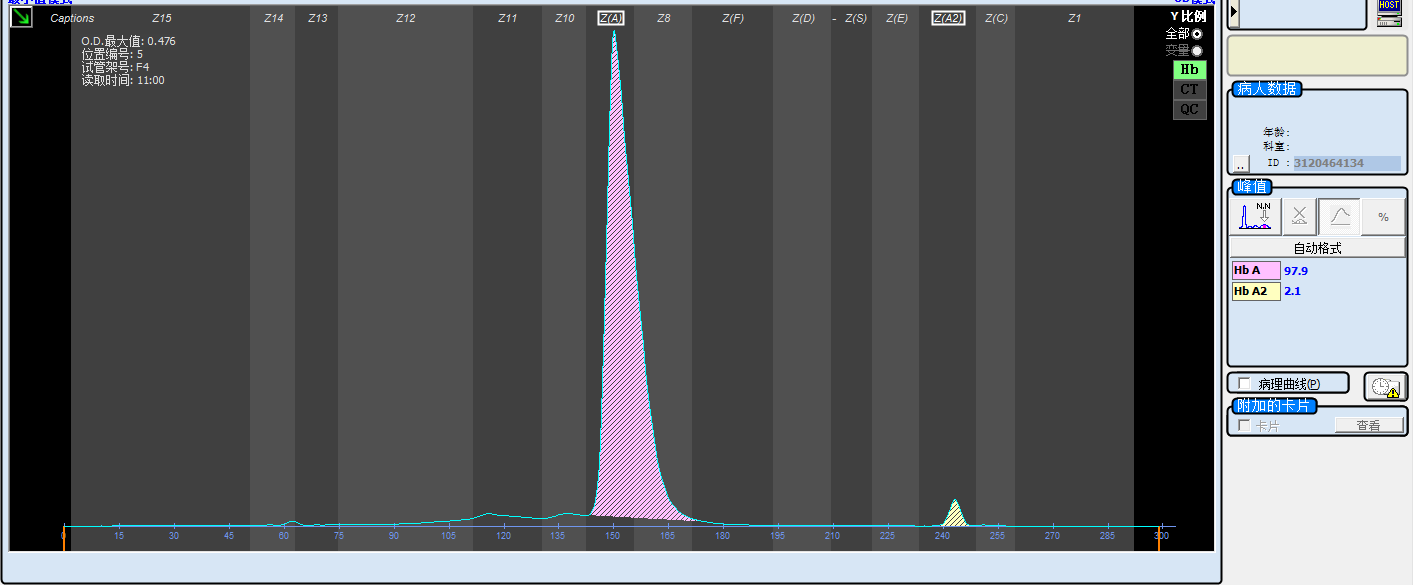

Supplement: S2 Fig — (ZIP) [file pone.0329365.s002.zip › S2_Fig25.png]

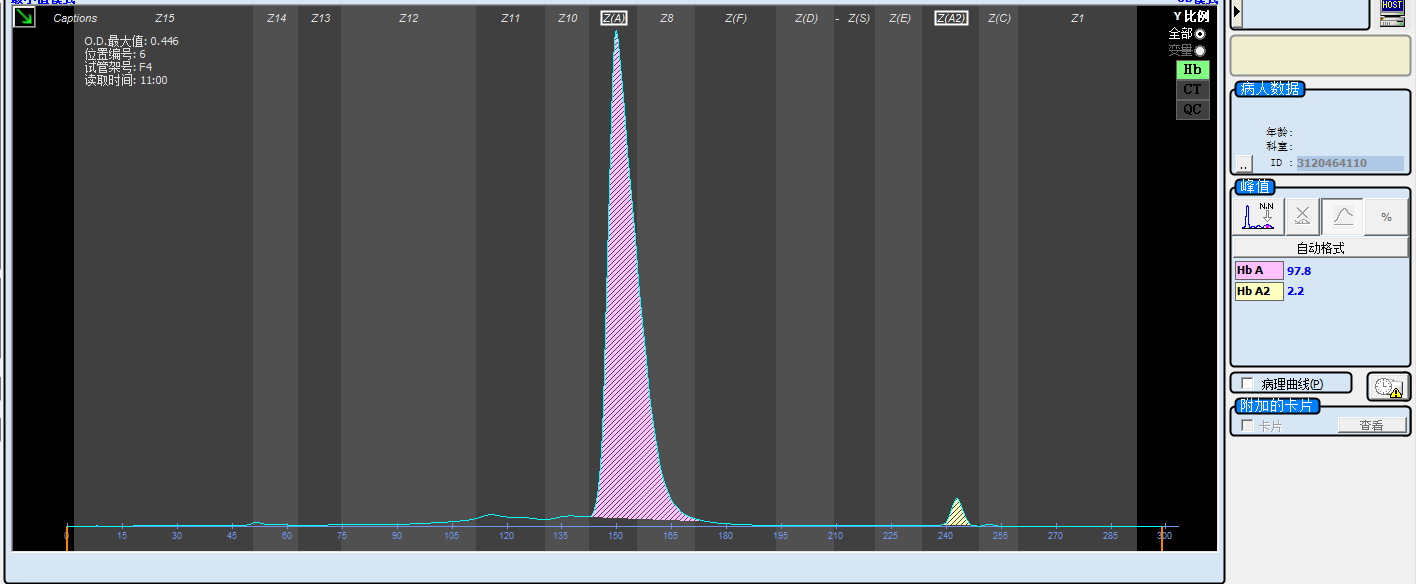

Supplement: S2 Fig — (ZIP) [file pone.0329365.s002.zip › S2_Fig26.png]

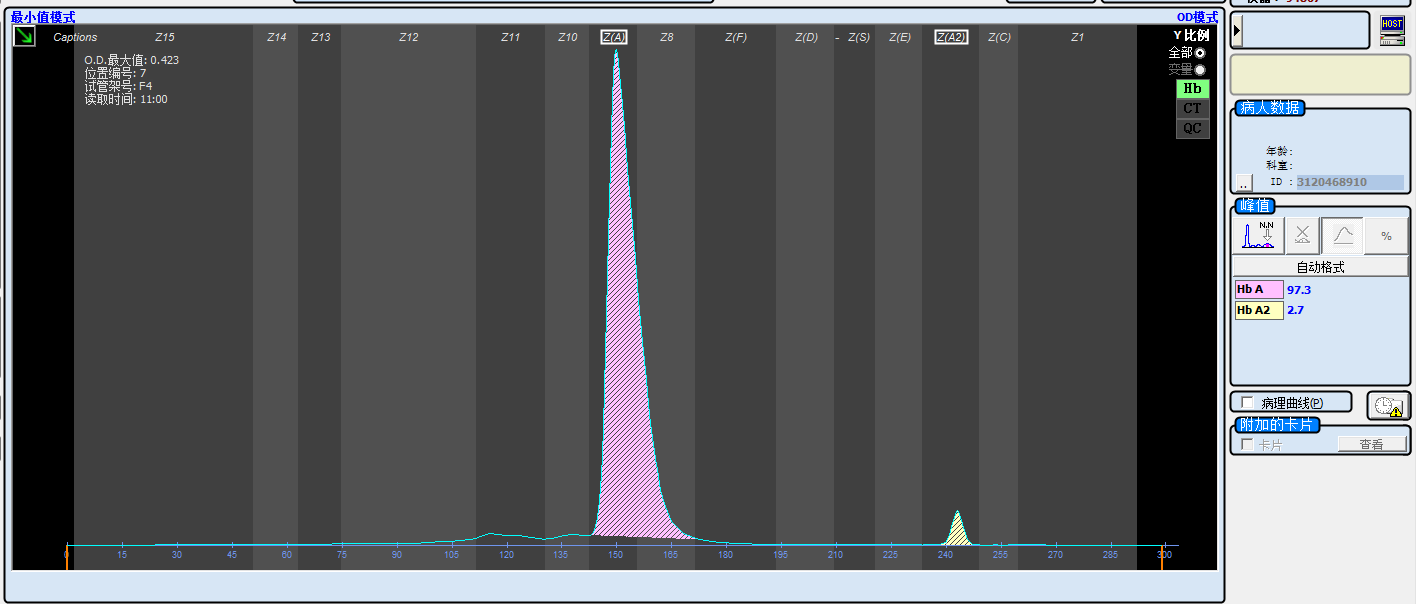

Supplement: S2 Fig — (ZIP) [file pone.0329365.s002.zip › S2_Fig27.png]

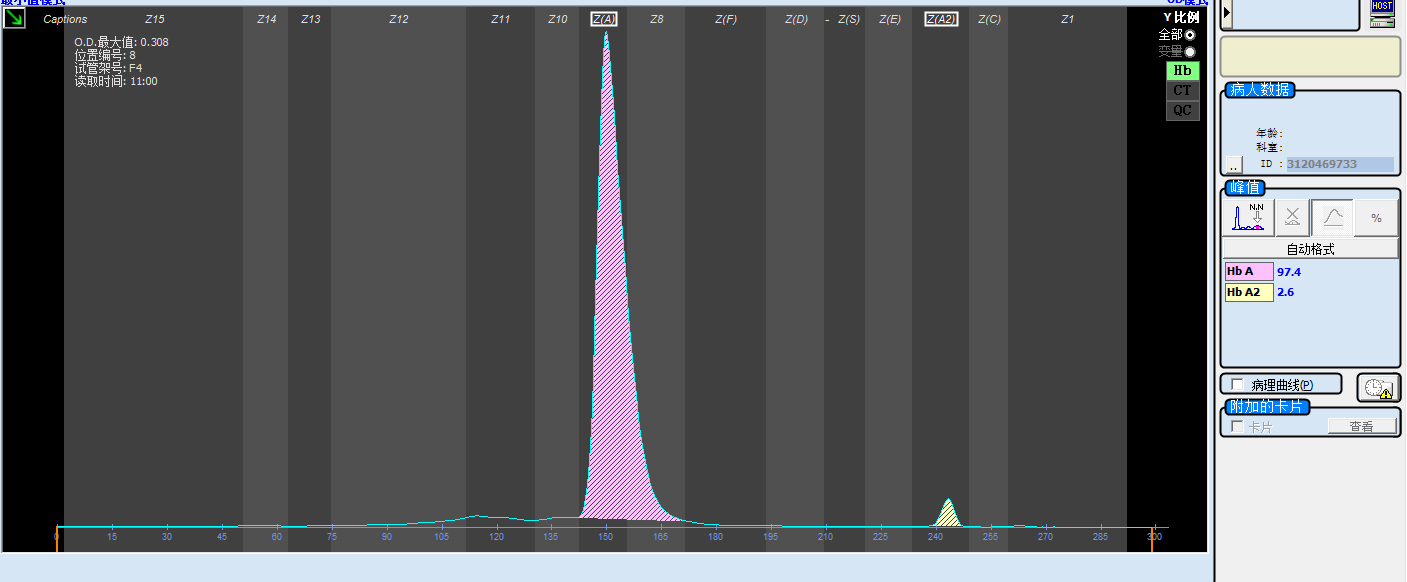

Supplement: S2 Fig — (ZIP) [file pone.0329365.s002.zip › S2_Fig28.png]

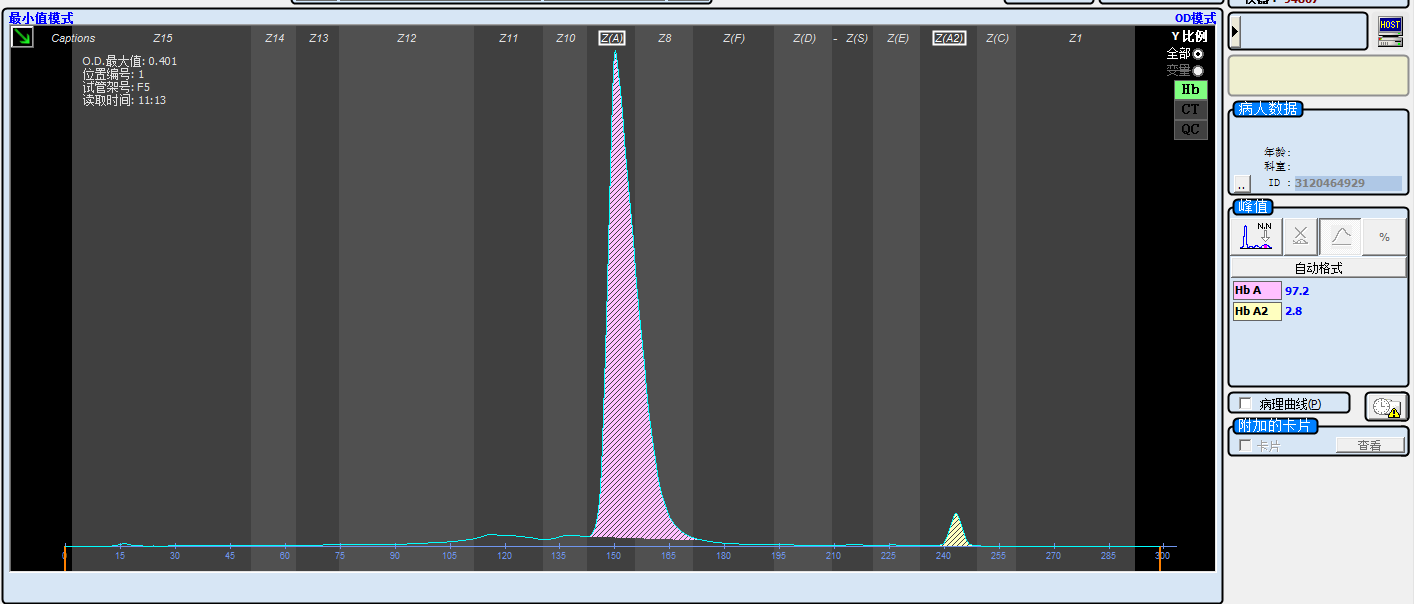

Supplement: S2 Fig — (ZIP) [file pone.0329365.s002.zip › S2_Fig29.png]

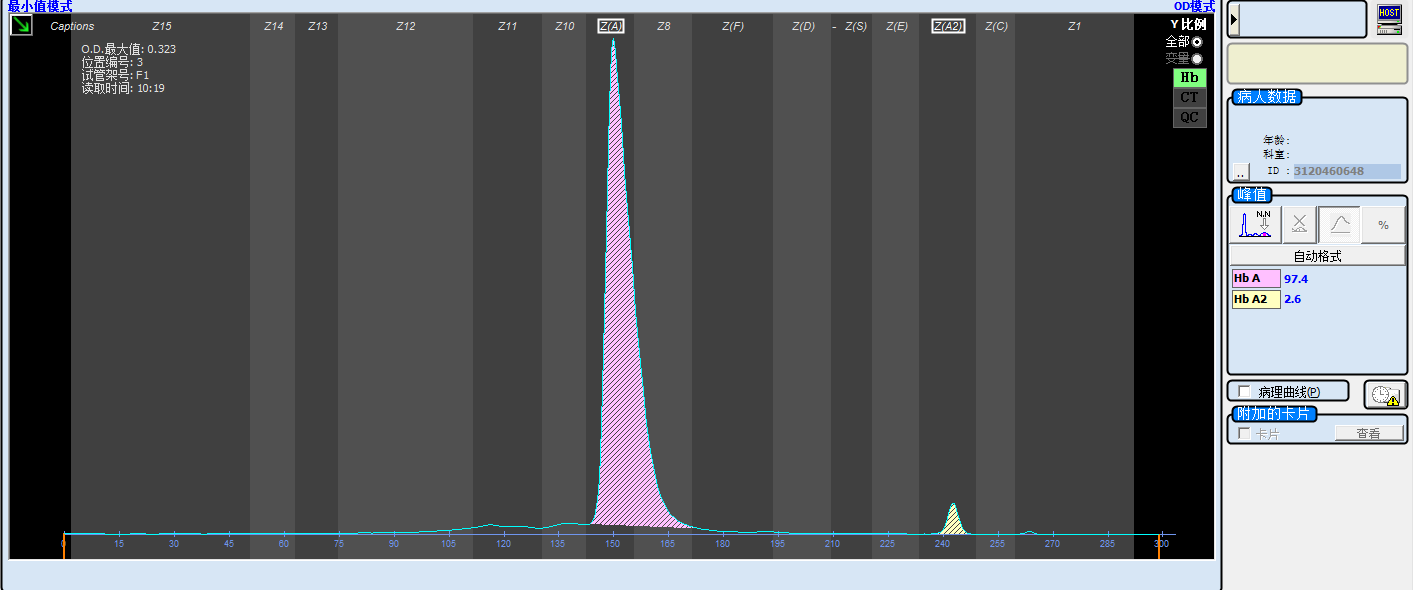

Supplement: S2 Fig — (ZIP) [file pone.0329365.s002.zip › S2_Fig3.png]

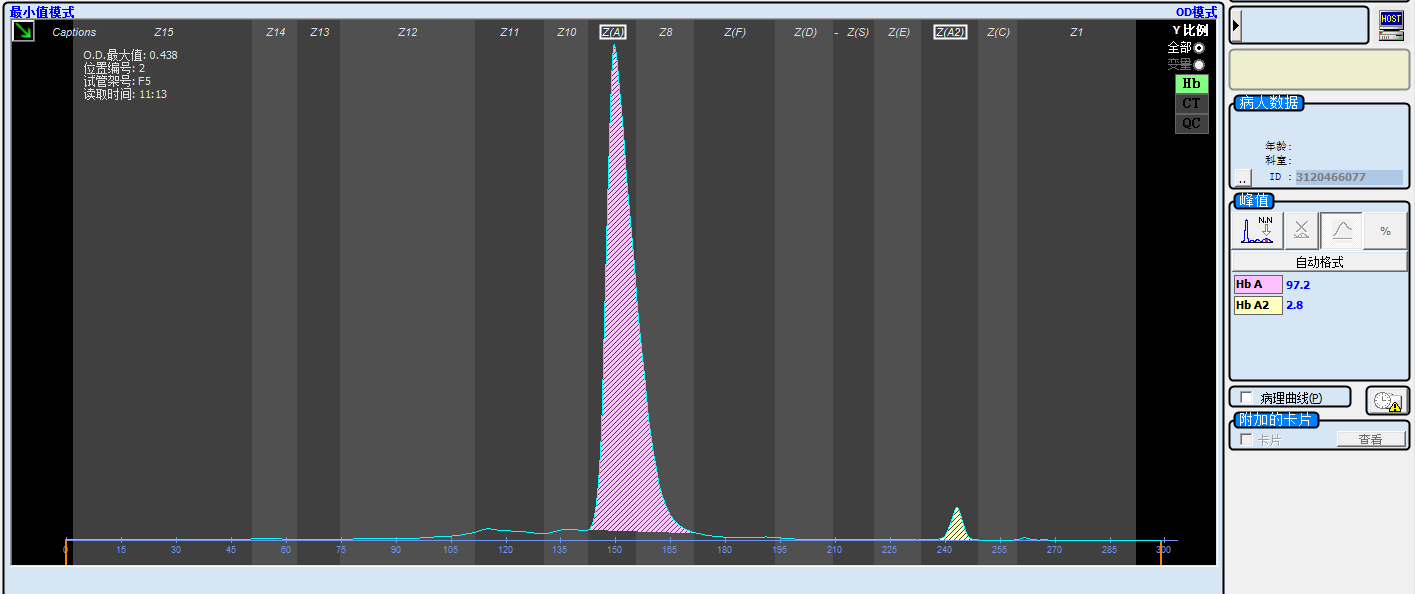

Supplement: S2 Fig — (ZIP) [file pone.0329365.s002.zip › S2_Fig30.png]

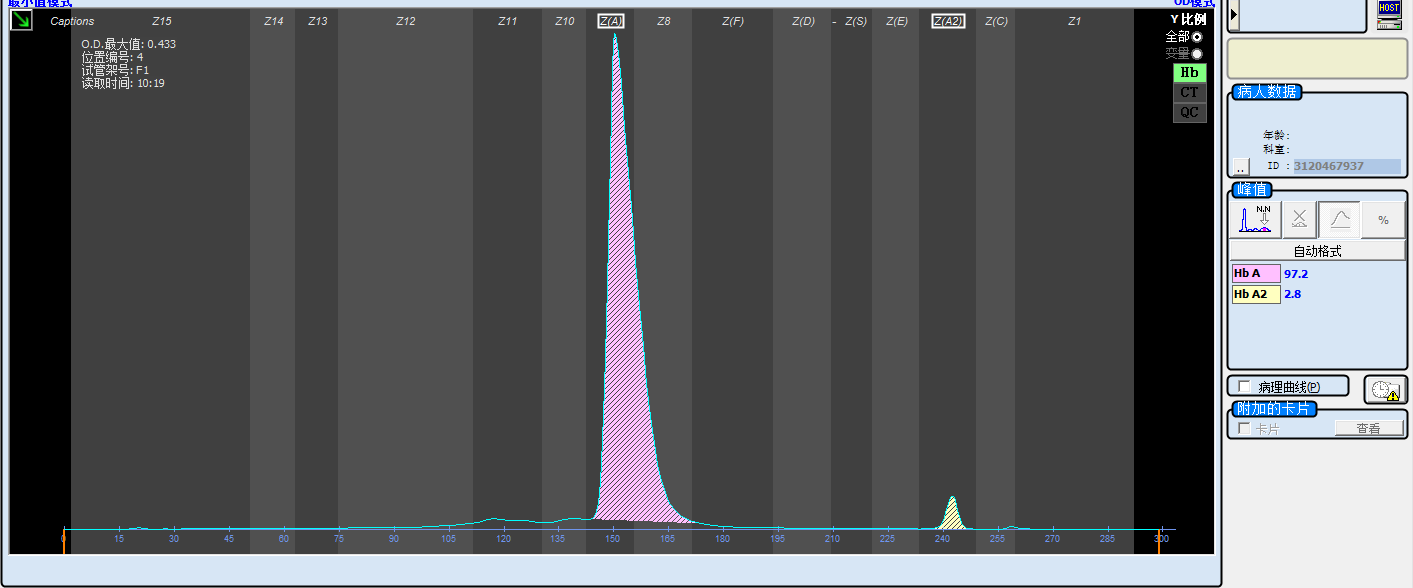

Supplement: S2 Fig — (ZIP) [file pone.0329365.s002.zip › S2_Fig4.png]

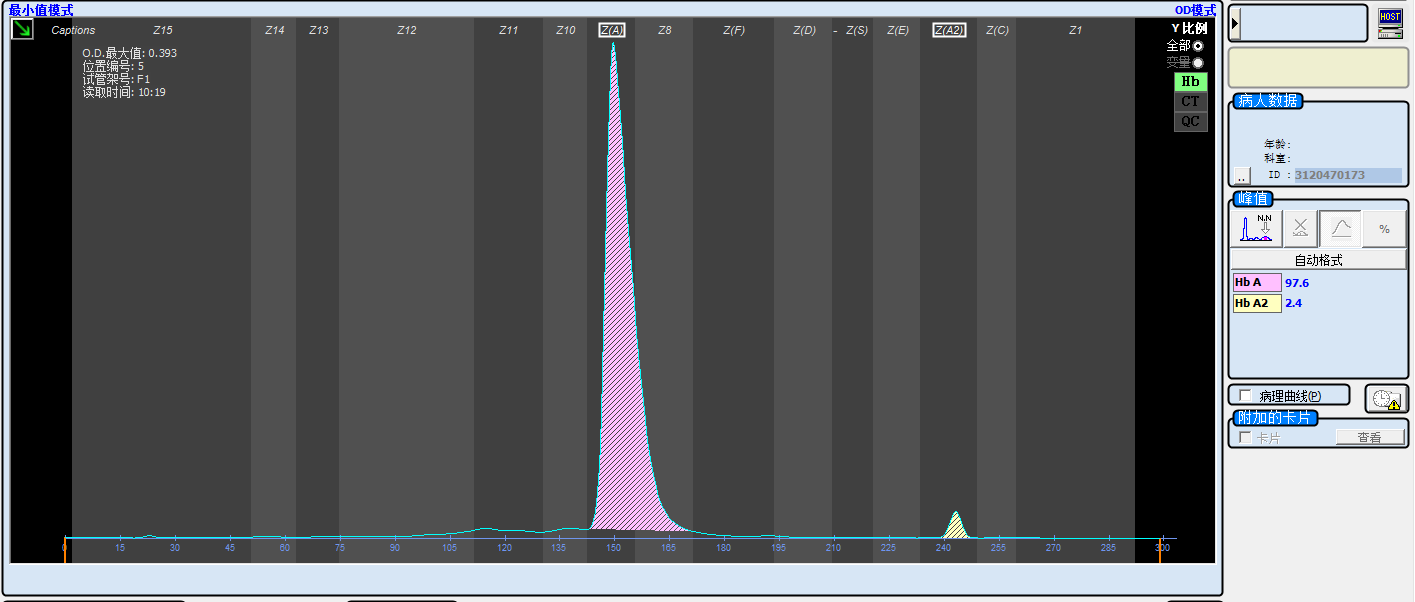

Supplement: S2 Fig — (ZIP) [file pone.0329365.s002.zip › S2_Fig5.png]

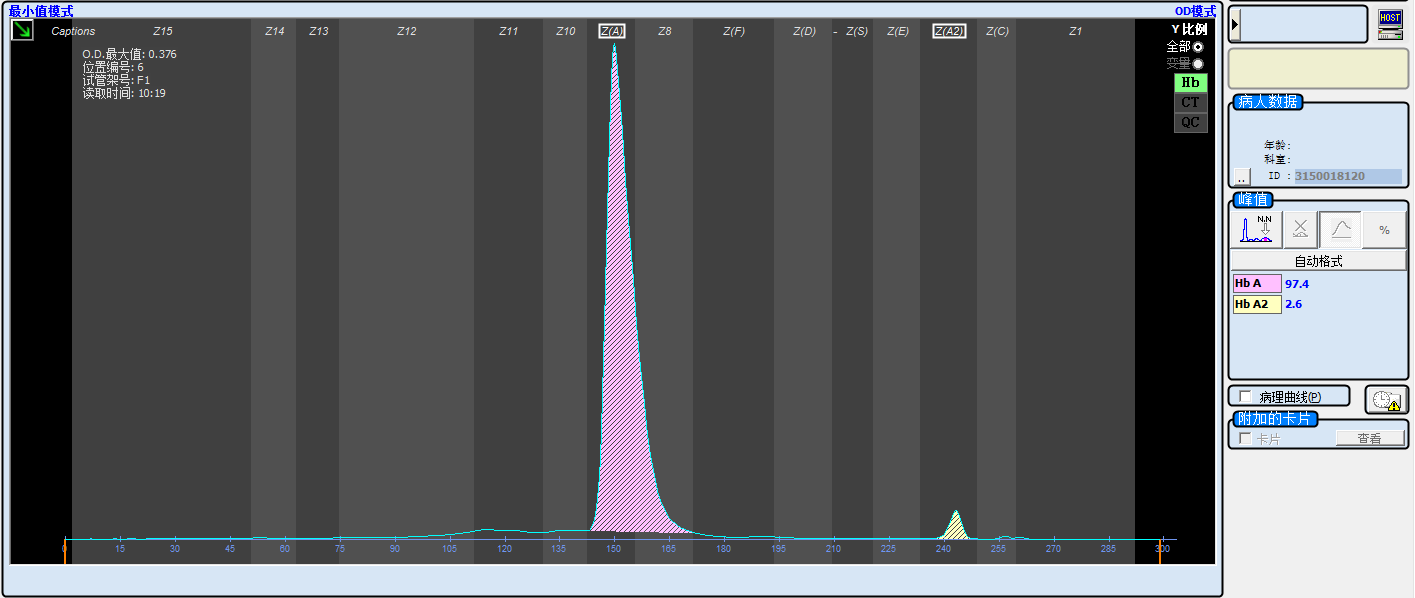

Supplement: S2 Fig — (ZIP) [file pone.0329365.s002.zip › S2_Fig6.png]

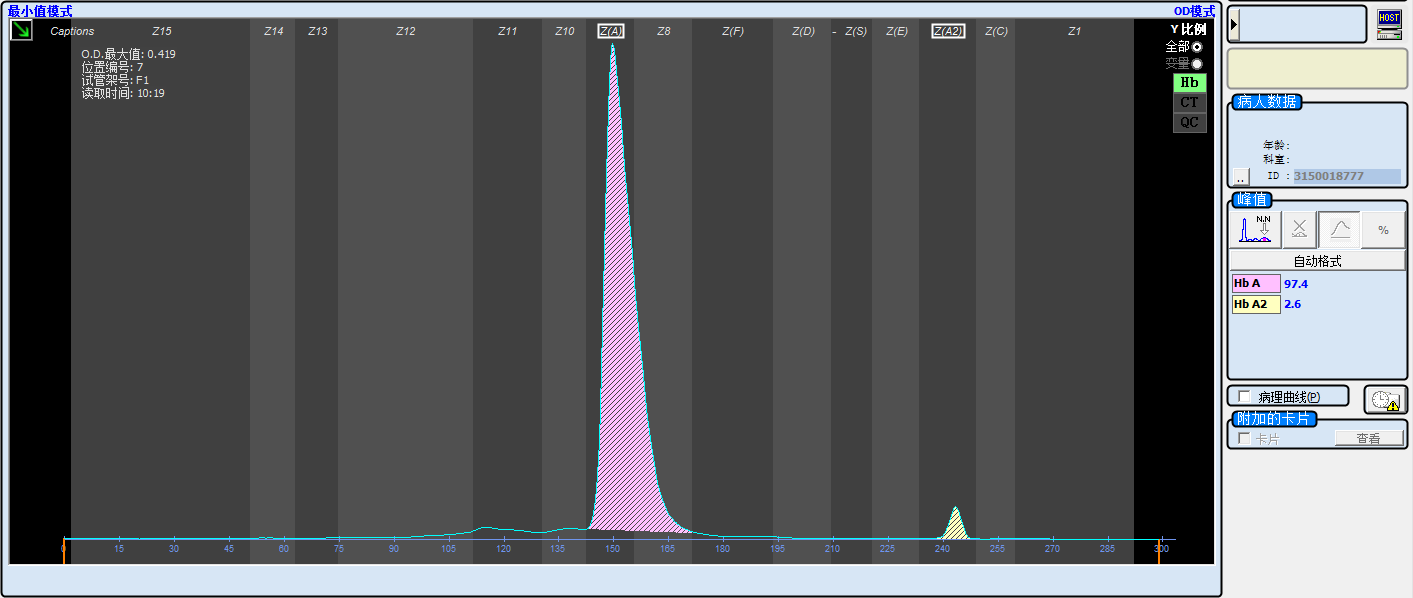

Supplement: S2 Fig — (ZIP) [file pone.0329365.s002.zip › S2_Fig7.png]

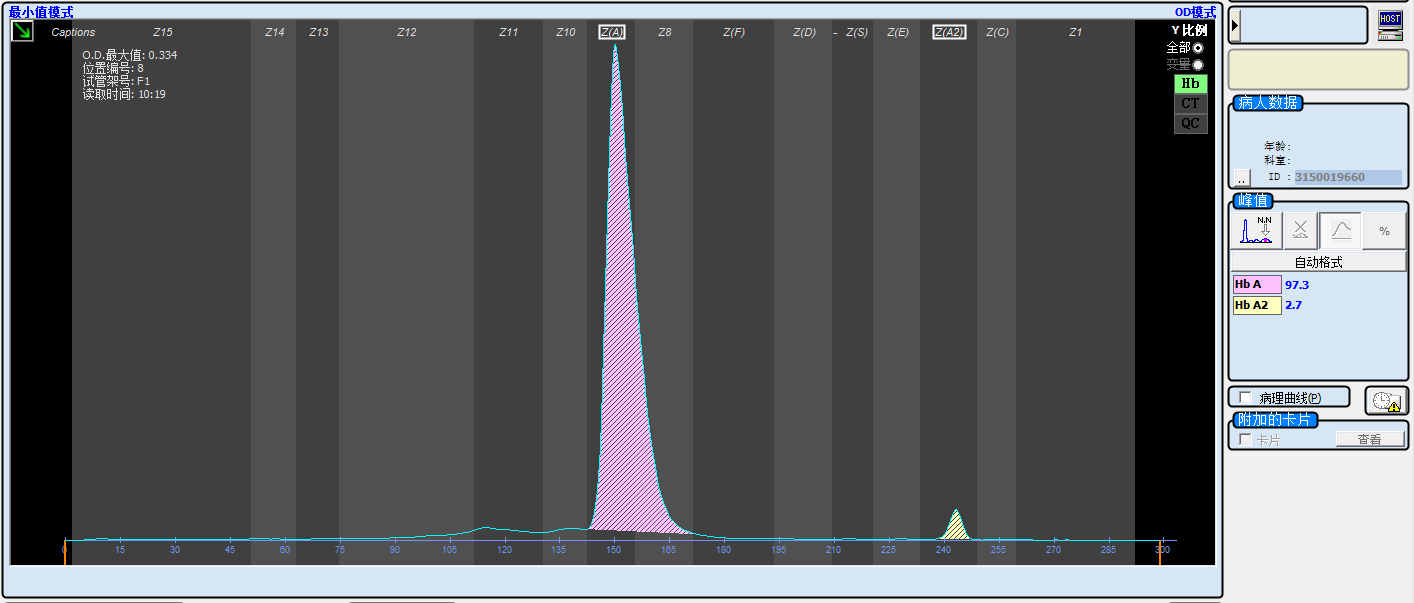

Supplement: S2 Fig — (ZIP) [file pone.0329365.s002.zip › S2_Fig8.png]

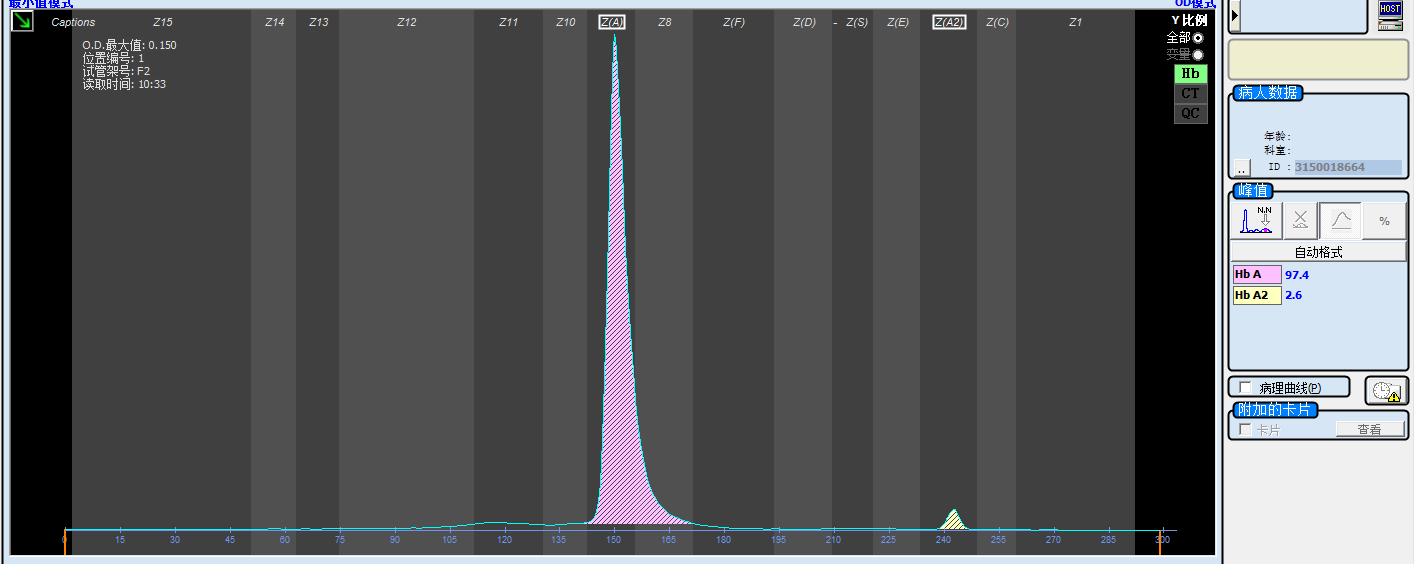

Supplement: S2 Fig — (ZIP) [file pone.0329365.s002.zip › S2_Fig9.png]
